# Design, synthesis, and anti-inflammatory potential of PROTAC drug molecules based on fondaparinux sodium

**DOI:** 10.3389/fbioe.2025.1597344

**Published:** 2025-07-07

**Authors:** Ruoxuan Wu, Tianji Zhang, Siran Zhao, Marco Maccarana, Jin-Ping Li, Chao Li, Hui Cao

**Affiliations:** ^1^ State Key Laboratory of Green Biomanufacturing, Beijing University of Chemical Technology, Beijing, China; ^2^ National Energy R&D Center for Biorefinery, Beijing University of Chemical Technology, Beijing, China; ^3^ Beijing Key Laboratory of Green Chemicals Biomanufacturing, Beijing University of Chemical Technology, Beijing, China; ^4^ Beijing Synthetic Bio-Manufacturing Technology Innovation Center, Beijing University of Chemical Technology, Beijing, China; ^5^ Division of Chemistry and Analytical Science, National Institute of Metrology, Beijing, China; ^6^ Key Laboratory of Chemical Metrology and Applications on Nutrition and Health for State Market Regulation, National Institute of Metrology, Beijing, China; ^7^ Department of Medical Biochemistry and Microbiology, University of Uppsala, Uppsala, Sweden; ^8^ Beijing Advanced Innovation Centre for Soft Matter Science and Engineering, Beijing University of Chemical Technology, Beijing, China

**Keywords:** fondaparinux sodium (FS), proteolysis targeting chimera (PROTAC), surface plasmon resonance (SPR), cytokine suppression, anti-inflammatory

## Abstract

**Introduction:**

In this study, we used an approach by conjugating Fondaparinux Sodium (FS) with selected drugs to generate proteolysis-targeting chimeras (PROTACs).

**Methods:**

By applying bioprocess engineering principles, the direct amidation reaction was optimized –through precise control of pH, substrate ratios, and solvent selection –to reliably produce high‐purity (>99%) PROTAC molecules on a scalable platform. Surface plasmon resonance (SPR) analysis demonstrated that the synthesized PROTACs exhibit micromolar binding affinities (KD ≈ 10^–6^ M) toward inflammatory mediators RANTES (CCL5) and interleukin-6 (IL-6). In vitro assays using peripheral blood mononuclear cells (PBMCs) revealed that two candidate compounds (Product 6 and Product 10) significantly inhibited lipopolysaccharide (LPS)‐induced interleukin‐1β (IL‐1β) release in a concentration-dependent manner, while FS and the drugs alone had no effect.

**Results:**

High-purity (>99%) PROTAC molecules were produced on a scalable platform. The synthesized PROTACs demonstrated micromolar binding affinities (KD ≈ 10^–6^ M) toward RANTES (CCL5) and IL-6. Two candidate compounds (Product 6 and Product 10) significantly inhibited LPS-induced IL-1β release in PBMCs in a concentration-dependent manner; FS and the drugs alone showed no effect.

**Discussion:**

These findings not only provide an innovative strategy for targeting “undruggable” proteins but also establish a robust, scalable process for the production of PROTAC‐based anti-inflammatory agents.

## 1 Introduction

Proteolysis-targeting chimeras (PROTACs) are bifunctional molecules comprising a ligand for the protein of interest (POI), an E3 ubiquitin ligase–recruiting moiety, and a flexible linker (Zhong et al., 2024). By simultaneously engaging the target protein and an E3 ligase, PROTACs induce formation of a ternary complex that promotes ubiquitination and subsequent 26S proteasomal degradation of the POI (Chen et al., 2024). This “event-driven” mechanism enables degradation of traditionally “undruggable” targets, such as transcription factors lacking well-defined active sites (Wang et al., 2024). Several CRBN- and VHL-based PROTACs have advanced into clinical trials for oncology and inflammatory indications, demonstrating the translational potential of this modality (Vetma et al., 2024; Guedeney et al., 2023; Békés et al., 2022; Krieger et al., 2023; He et al., 2024; Tan et al., 2025).

Despite these advances, three primary challenges hinder PROTAC development. First, their large molecular weight and high polarity often result in poor aqueous solubility and limited cell permeability, leading to rapid clearance and low oral bioavailability *in vivo* (Sincere et al., 2023; Abeje et al., 2024). Second, at elevated concentrations PROTACs may form non-productive binary complexes with either the POI or the E3 ligase, thereby reducing degradation efficiency (Ciulli and Trainor, 2021). Third, effective *in vivo* delivery frequently depends on specialized formulations or carrier systems.

In inflammatory diseases, stimulated by pathogens or autoimmune triggers, induces release of pro-inflammatory cytokines such as IL-6 and CCL5, amplifying the inflammatory cascade. Current therapies—for example, IL-6 receptor antibodies (e.g., tocilizumab) or small-molecule signaling inhibitors—neutralize specific mediators but do not catalytically remove them, and may incur immunogenicity or off-target toxicity (Liu et al., 2022; Howard et al., 2025).

To address these limitations, numerous strategies have been explored, including linker optimization (Jeong et al., 2025), nano- or lipid- and polymer-based carrier systems (Syahputra et al., 2025), and peptide- or glycan-based delivery vehicles (Todaro et al., 2023). Among these, heparin-like oligo- or polysaccharides bearing multiple sulfate and carboxyl groups exhibit high anionic character and water solubility, enabling strong electrostatic interactions with cationic residues (e.g., Arg, Lys) on protein surfaces and offering a promising scaffold for PROTAC design (Shute, 2023).

Glycan-based carriers—such as heparin, hyaluronic acid, dextran, chitosan, and engineered oligosaccharides—have gained attention for tissue-targeted or controlled release applications. These molecules are biocompatible and biodegradable, and can load drugs via electrostatic or covalent linkages (Sun et al., 2020; Kurczewska, 2022). For example, heparin derivatives formulated into nanoparticles or hydrogels improve drug solubility and stability, and their sulfate groups can neutralize extracellular toxins and cytokines without significant anticoagulant activity, thereby exerting anti-inflammatory effects while serving as a delivery platform (Shute, 2023).

Building on these insights, we conjugated the highly sulfated pentasaccharide Fondaparinux Sodium (FS) to selected small-molecule ligands. Incorporation of FS is expected to enhance the solubility and biocompatibility of the PROTAC scaffold, strengthen extracellular target engagement through multivalent electrostatic interactions, and facilitate efficient recruitment of the E3 ligase to promote proteasomal degradation (Liu et al., 2022) Compared to flexible hydrophilic carriers such as polyethylene glycol (PEG) or polysialic acid, the rigid FS backbone imposes defined stereochemistry and multivalent sulfate presentation, reducing conformational entropy loss and improving binding specificity (Syahputra et al., 2025). In contrast, PEG’s random-coil structure may compromise precise spatial orientation of the target–E3 complex (Sun et al., 2020), and polysialic acid’s high hydration and size can impede cellular uptake and increase nonspecific binding (Kurczewska, 2022).

To validate this approach, we selected three cereblon-binding ligands—pomalidomide, lenalidomide, and thalidomide—for 1:1 conjugation with FS. We optimized multiple amidation routes to avoid multi-substitution of FS hydroxyls and ensure stoichiometric coupling. The resulting PROTACs were characterized by surface plasmon resonance (SPR) to determine binding kinetics with IL-6 and CCL5, and their anti-inflammatory efficacy was evaluated in LPS-stimulated human peripheral blood mononuclear cells by measuring cytokine release.

## 2 Materials and methods

### 2.1 Reagents and instruments

All chemicals—including FS, E3 ligase ligand–linker conjugate, EDCHCl, NHS, and 1-hydroxybenzotriazole (HOBt)—were of analytical grade and purchased from Sigma-Aldrich and Thermo Fisher Scientific. Reaction solvents, such as N,N-dimethylformamide (DMF) and acetonitrile (ACN), were dried using molecular sieves before use. Nuclear Magnetic Resonance (NMR) analyses were performed on a Bruker 600 MHz spectrometer, and mass spectrometry was carried out using a Thermo LTQ-Orbitrap VELOS PRO system. Purification was executed on an AKTA protein purification system (Cytiva), and SPR experiments were conducted on a Biacore 8K^+^ system.

### 2.2 Chemical synthesis and reaction conditions

#### 2.2.1 Reaction design

To simplify the conventional protection–deprotection procedures, a direct amidation strategy without protecting groups was adopted. The carboxyl group of FS was used as the reaction site and directly coupled with amine-containing E3 ligase ligand–linker conjugate (including Pomalidomide-C7-NH_2_, Lenalidomide-C5-NH_2_, and Thalidomide-NH-C5-NH_2_) to construct bifunctional PROTAC molecules (Table 1). We selected pomalidomide, lenalidomide, and thalidomide E3 ligase ligands based on their well-characterized cereblon-binding affinities (K_D_ CRBN ≈ 0.5–1 μM, 0.5–1 μM, and 1–2 μM, respectively) and published differences in degradation efficacy in previous PROTAC platforms.

**TABLE 1:** The structural depiction of product 6, 8, 10.

Compound	Scaffold	E3Ligase ligand	Linker length	Yield (%)	Purity (%)
Product 6	FS	Pomalidomide	C7	68	>99
Product 8	FS	Lenalidomide	C5	71	>99
Product 10	FS	Thalidomide	NH-C5	69	>99

#### 2.2.2 Reaction conditions

To a mixture of FS (20 mg, 1.0 equiv.) in ACN/H_2_O (5:2, v/v, 1.40 mL) was added E3 ligase ligand–linker conjugate (10.0 equiv.), EDCHCl (10.0 equiv.), NHS (10.0 equiv.), and HOBt (10.0 equiv.) at room temperature. The pH was adjusted to 8.5 with 0.1 M NaHCO_3_, and the reaction was stirred for 6 h. Afterward, the mixture was subjected to DEAE-Sephadex weak anion exchange chromatography (gradient elution with 0.05–1.5 M NaCl), followed by dialysis (MWCO 1000 Da) and lyophilization, affording the crude product. The structure was confirmed by LC-MS and ^1^H NMR. Yields were initially estimated by normalizing the total ion chromatogram (TIC) peak areas and subsequently verified by comparing the actual yield to the theoretical value (calculated from the initial molar quantities). Reaction parameters (pH, reagent ratios, etc.) were systematically optimized (see Figures 3–5).

#### 2.2.3 Surface plasmon resonance (SPR) experiments

SPR was used to evaluate the binding affinity between the PROTAC molecules and the target proteins. Target proteins CCL5 and IL-6 were immobilized on a CM5 sensor chip via amine coupling, with unreacted sites blocked using ethanolamine (pH 8.5). The PROTAC molecules were injected at concentrations ranging from 0.38 µM to 12.50 µM in PBS-P running buffer (0.02 M phosphate buffer, 137 mM NaCl, 2.7 mM KCl, 0.2% DMSO, 0.05% P20, pH 7.4). Each injection lasted 120 s (association phase) followed by a 300-s dissociation phase at a flow rate of 30 μL/min. Data were analyzed using Biacore Insight Evaluation Software with a 1:1 binding model to determine K_D_ values. In addition to the 1:1 Langmuir model, we tested bivalent-analyte and heterogeneous-ligand models. Both alternatives yielded substantially larger residuals and poorer overall fits, confirming the 1:1 Langmuir model as the most suitable for our sensorgram data. The results demonstrated that the PROTAC molecules exhibited significantly stronger binding to the target proteins compared to FS.

#### 2.2.4 Anti-inflammatory activity assays

##### 2.2.4.1 Cytokine release in peripheral blood mononuclear cells (PBMCs)

Human PBMCs were isolated from buffy coats (procured from Uppsala University Hospital, Sweden) using Ficoll density gradient centrifugation and stored at −150°C. Thawed PBMCs were cultured in Dulbecco’s Modified Eagle Medium (DMEM) supplemented with 10% fetal bovine serum (FBS) and 5% PeSt in a CO_2_ incubator. Cells were seeded in 24-well plates (0.5–1 × 10^6^ cells/well in 1 mL medium). PROTAC analogs and FS were added at 10 μM, 1 μM, and 100 nM. Two setups were employed: one receiving only the compounds, and another in which LPS (20 ng/mL) was added 2 h after compound treatment. After 24 h, supernatants were collected for cytokine quantification by ELISA.

#### 2.2.5 Statistical analysis

All quantitative data are presented as mean ± SD. Sample size for each assay was n = 3 biological replicates. For multi-group comparisons, one-way ANOVA followed by Tukey’s *post hoc* test was used; for two-group comparisons, two-tailed, unpaired Student’s t-test was applied. *P* < 0.05 was considered significant.

## 3 Results and discussion

### 3.1 Molecular design and structural characterization

The PROTAC molecules were constructed using FS as the core scaffold and coupled with E3 ligase ligands via a direct amidation reaction. The FS molecule, which is rich in sulfate and carboxyl groups, possesses a strong anionic character that promotes multiple electrostatic interactions with positively charged regions on target proteins. This interaction significantly enhances binding affinity and complex stability, thereby extending the range of proteins that are traditionally considered “undruggable” (Antermite et al., 2023). In this design, the carboxyl group—selected for its high reactivity and ability to form stable amide bonds—ensures *in vivo* stability and circumvents the harsh conditions and hydrolysis issues associated with hydroxyl groups (Crowley, 2022). Moreover, the excellent water solubility and biocompatibility of FS improve *in vivo* distribution and bioavailability, while its rigid structure and long half-life ensure sustained activity under physiological conditions, facilitating efficient recruitment of the E3 ligase for target degradation.

Characterization by ^1^H NMR and LC-MS (see Supplementary Tables S1, S2; Supplementary Figures S3–S6) confirmed that the molecular ion peaks and fragmentation patterns of the target products were as expected. Preliminary yield estimations based on TIC peak normalization were in good agreement with theoretical values, demonstrating effective control over product selectivity under optimized conditions.

### 3.2 Chemical synthesis and condition optimization

#### 3.2.1 Optimization of the catalytic system

**FIGURE 1:**
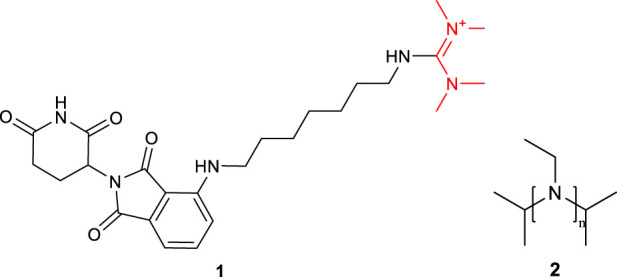
Structural formulas of by-product 1 and polymers 2 formed during the coupling reaction using HBTU and i-Pr_2_NEt.

Initially, HBTU was employed as the coupling reagent for the amidation reaction between FS and the ligand (Table 3, entry A0). However, LC-MS analysis revealed that the desired PROTAC was not obtained; instead, a large amount of 1 and 2 formed (Figure 1; Supplementary Figures S1, S2), primarily due to the high reactivity of HBTU leading to direct reactions with the amine. To mitigate these non-specific side reactions, we subsequently adopted the EDC/NHS system. Under H_2_O/ACN (2:1) conditions at room temperature for 4 h, LC-MS confirmed the formation of PROTAC-like molecules, yet the overall yield remained low (Table 3, entry A1), accompanied by intermediates 3 or 4. These results underscore the importance of selecting an appropriate catalytic system to improve both reaction selectivity and yield. Further optimization of reaction parameters is warranted to reduce side reactions.

#### 3.2.2 Reaction condition optimization

To improve the yield and refine the synthesis method, various parameters were systematically optimized—including the ratio of coupling reagents, solvent composition, reactant stoichiometry, and the pH during carboxyl activation and amine coupling. Detailed experimental data are presented in Tables 2, 3.

**TABLE 2:** Synthesized molecular structures of PROTAC-like molecules.

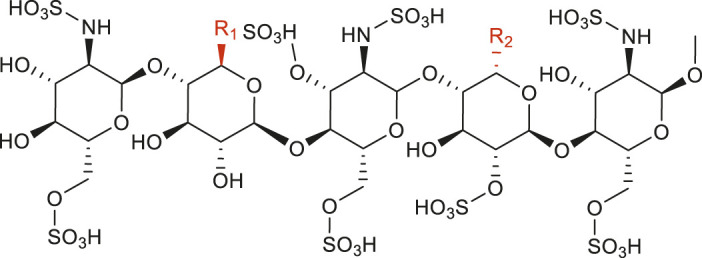			
Compound	R1	R2	Yield%
By-product 5 (a, b)	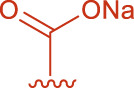	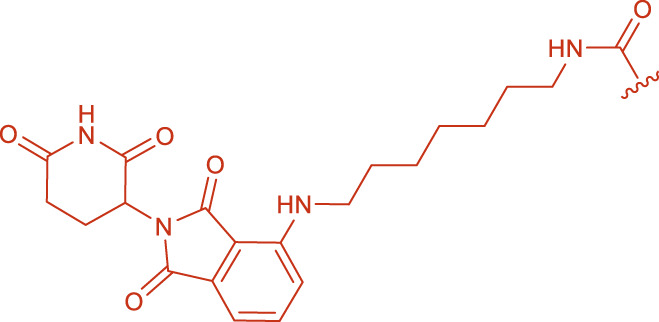	34% (A8)
Product 6	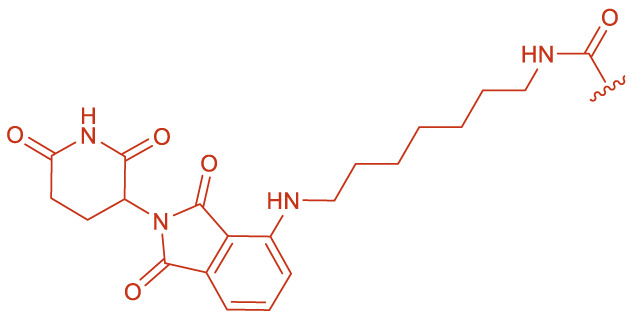	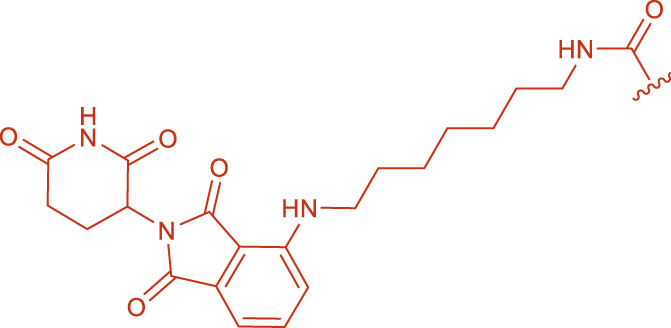	68% (A15)
By-product 7 (a, b)	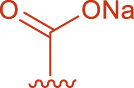	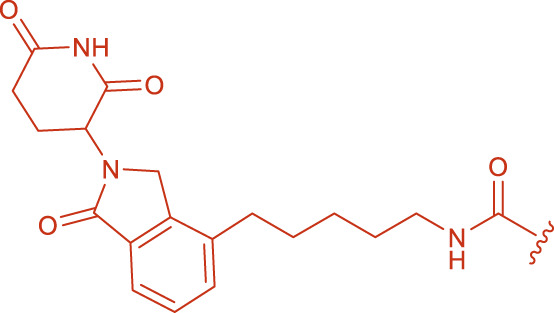	36% (A8^a^)
Product 8	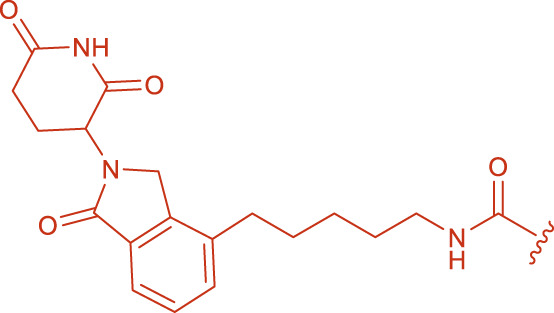	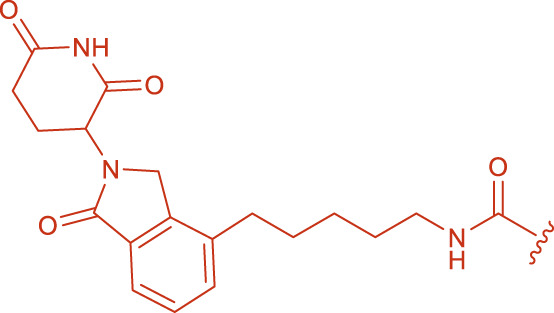	71% (A15^a^)
By-product 9 (a, b)	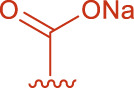	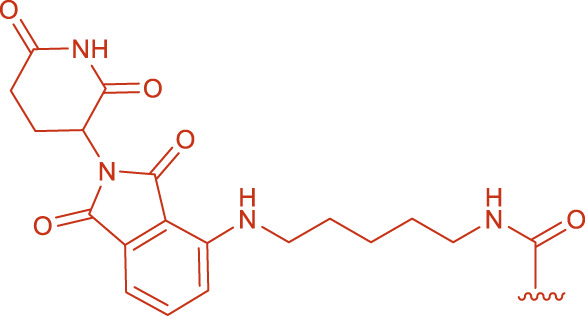	38% (A8^b^)
Product 10	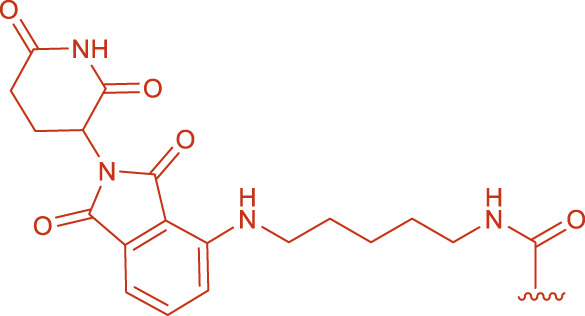	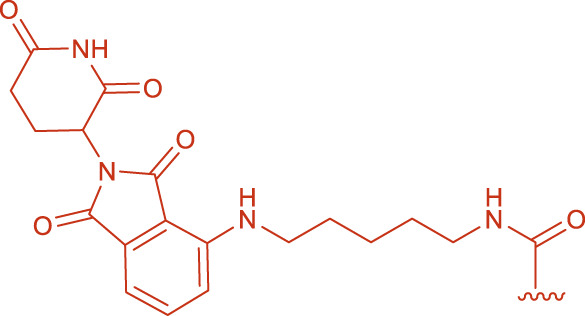	69% (A15^b^)

a, b refer to regioisomers where the R1 and R2 groups are swapped, and the yield represents the combined yield of both (as they are difficult to separate).

^a^ Only the Pomalidomide molecule was replaced with Lenalidomide under otherwise identical conditions.

^b^ Only the Pomalidomide molecule was replaced with Thalidomide under otherwise identical conditions.

**TABLE 3:** The yield (%) of product 6 under the different reaction conditions.

Number	EDC⋅HCl (equiv.)	HOBt (equiv.)	NHS (equiv.)	pH	FS: Ligand	Product 6 yields (%)^a^	Byproduct 5 (%)
A0^b^	–	–	–	5.50 to 5.50	1:1	ND	ND
A1	1.0	–	–	5.50 to 5.50	1:1	ND	trace
A2	1.0	1.2	–	5.10 to 5.50	1:1	trace	4
A3	2.0	2.4	2.0	5.50 to 5.50	1:1	ND	18
A4	2.0	2.4	2.0	5.50 to 7.50	1:1	12	34
A5	2.0	2.4	2.0	5.50 to 7.89	1:1	12	34
A6	2.0	2.4	2.0	3.84 to 7.60	1:2	6	31
A7	2.0	2.4	2.0	5.11 to 7.66	1:2	28	32
A8	4.0	4.8	4.0	5.23 to 7.63	1:4	30	32
A9	10.0	10.0	10.0	5.50 to 5.50	1:10	trace	ND
A10	10.0	10.0	10.0	5.50 to 6.04	1:10	10	4
A11	10.0	10.0	10.0	5.50 to 6.54	1:10	trace	ND
A12	10.0	10.0	10.0	5.50 to 7.00	1:10	38	22
A13	10.0	10.0	10.0	5.50 to 7.54	1:10	25	28
A14	10.0	10.0	10.0	5.50 to 8.12	1:10	61	16
A15	10.0	10.0	10.0	5.50 to 8.50	1:10	68	12
A16	10.0	10.0	10.0	5.50 to 9.00	1:10	29	11

^a^ Yield was defined as the proportion of Product 6 relative to the total mass of substances in the final reaction mixture (the total mass of the material minus excess E3 ligase ligand–linker conjugate (ligand)) and was calculated using the corresponding peak area in MS spectrum.

^b^ HBTU (1.1 eq) was added. In this table, unused reagents are indicated by “-”.

##### 3.2.2.1 Adjustment of the coupling reagent ratios

**FIGURE 2:**
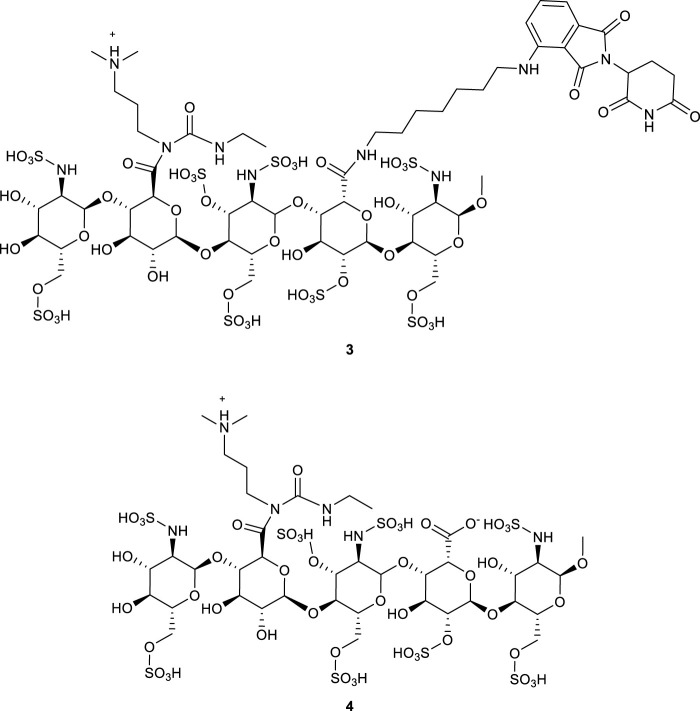
Structural formulas of intermediate 3 and intermediate 4.

EDC activates carboxyl groups by forming an O-acylisourea intermediate, which under ideal conditions is attacked by an amine to form an amide bond. However, if the amine’s nucleophilicity is insufficient or the reaction conditions (e.g., pH, temperature) are not optimal, the intermediate may rearrange to form a stable but inactive N-acylurea by-product (Crowley, 2022; Neri-Cruz et al., 2023). In the reaction between EDC and FS, the relatively low reactivity of the carboxyl groups in intermediates 3 or 4 (Figure 2) led to slow amidation and accumulation of the O-acylisourea intermediate, which then underwent rearrangement. To mitigate this, NHS was introduced; its presence enhanced the coupling efficiency by forming a more stable activated ester and reducing side reactions. Under pH 7.5, a yield of 12% was obtained (Table 3, entry A4).

##### 3.2.2.2 Influence of pH on the reaction

**FIGURE 3:**
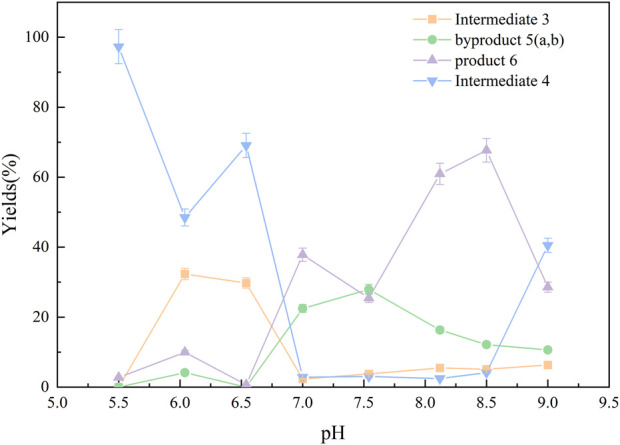
Yields (%) of each substance under different pH conditions. Amide coupling was evaluated from pH 5.5 to 9.0. Optimal yields for the desired di-amidation product 6 were achieved at pH 8.0–8.5, reaching 61% (pH 8.12) and 68% (pH 8.50), compared to 12% at pH 7.5. Acidic conditions (< pH 6.0) gave <1% yield, while overly alkaline conditions (> pH 8.5) led to increased side reactions and reduced efficiency.

pH critically affects the distribution and selectivity of product (Figure 3). Under acidic conditions (pH < 6), the carboxyl groups of FS exist primarily as non-ionized–COOH (Panja and Adams, 2022)^,^ while the amine in Pomalidomide-C7-NH_2_ is protonated to–NH_3_^+^, reducing its nucleophilicity and leading to low amidation efficiency and increased by-product 5 formation. In the pH range of 6.0–6.5, intermediate 3 predominated (Table 3, entry A9-A16); below pH 7.0, the formation of intermediate 4 increased. However, at pH 7.0–8.5, formation of both intermediates was markedly reduced. Notably, the target bis-amidated product, Product 6, was optimally generated at pH 8.0–8.5. Under these conditions, FS’s carboxyl groups existed as–COO^−^ (favoring activation by EDC) and the amine remains unprotonated (–NH_2_), thus enhancing nucleophilicity. In contrast, at low pH the amine is protonated, and at pH > 9, hydrolysis and reduced reagent stability diminish efficiency (Chen et al., 2023).

##### 3.2.2.3 Optimization of substrate ratios

Because the by-products were regioisomers that complicated separation, the focus was shifted to increasing the proportion of Product 6 by optimizing the substrate ratio. The ratio of FS to Pomalidomide was gradually increased from 1:1 to 1:10. As shown in Table 3 (entry A3-A15), improper substrate ratios led to decreased selectivity and increased by-product 5 formation. With a 1:10 ratio, the yield of Product 6 improved markedly from a maximum of 28%–68%. This adjustment minimizes the formation of unreacted carboxyl groups and mono-amidated by-products, thereby enhancing overall reaction selectivity.

#### 3.2.3 Final purification of the products

**FIGURE 4:**
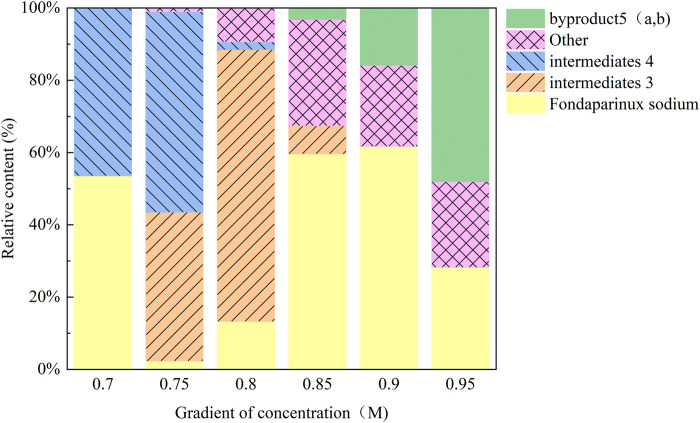
Changes in the relative content of each component under different elution salt concentrations. With increasing NaCl, intermediates 3 and 4 eluted first, followed by 5 (a, b) which reached 48.03% at 0.95 M. The target product 6 peak was obscured by co-eluting carboxylate polymers, indicating the need for tighter buffer control.

In the initial experiments, the crude mixture from condition A4 was purified using a DEAE-Sephadex anion exchange column on an AKTA system with a NaCl gradient (0.7 M–1.0 M). Analysis showed that (Figure 4), as the salt concentration increased, the elution order was roughly: intermediate 3 → intermediate 4 → other components → by-products 5. The by-products were most abundant in the 0.95 M NaCl fraction (48.03%). Notably, the characteristic peak for Product 6 was obscured by a strong signal corresponding to a sodium acetate polymer—likely derived from the buffer or as a byproduct—that competed for binding sites on the ion exchange resin. This interference indicated that further optimization of elution conditions or additional purification steps were necessary to remove salt polymers.

**FIGURE 5:**
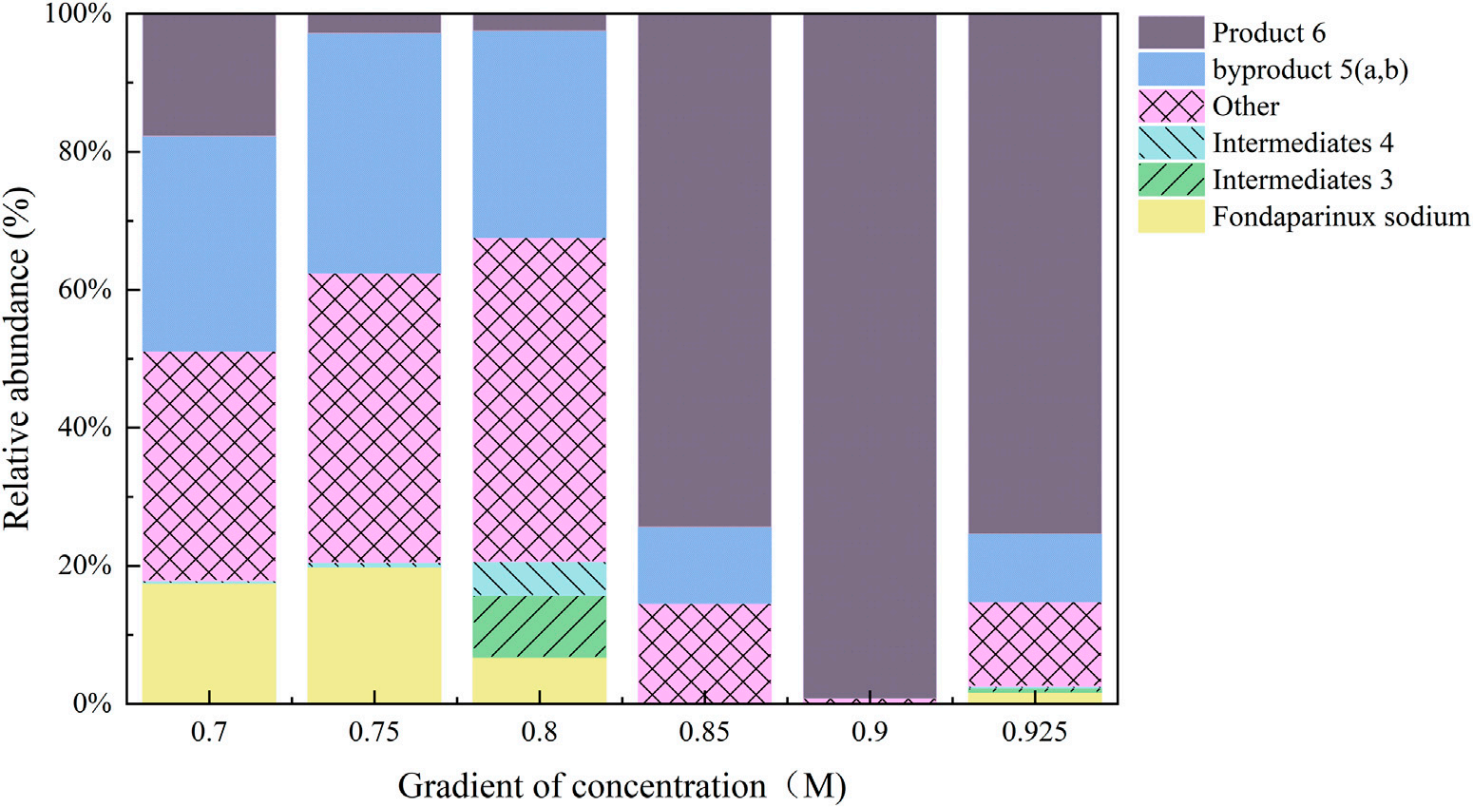
Purification condition optimization as a percentage of the sum of all substances (%). Using reaction mixture from condition A15 (pH 8.5, FS: ligand = 1:10, EDC/NHS) and raising buffer pH to 8.5, elution with 0.90 M NaCl yielded product 6 at 99.17% purity, effectively removing carboxylate polymer contaminants.

Subsequently, the optimized reaction mixture from condition A15 was purified using a 0.90 M NaCl eluent. Under these refined conditions, the purity of Product 6 was significantly enhanced, reaching up to 99.17% (Figure 5) according to the normalized peak area of the product and the by-products.

### 3.3 Surface plasmon resonance (SPR) analysis

To comprehensively assess the biological functionality of the PROTAC molecules, binding kinetics with the target proteins CCL5 and IL-6 were investigated using the Biacore 8K^+^ system. CCL5 and IL-6—key inflammatory mediators—were immobilized on a CM5 sensor chip (9000 RU) via amine coupling, and kinetic parameters were calculated using a 1:1 binding model.

#### 3.3.1 Overview of sensorgrams

During the association phase (120-s injection), the response units (RU) increased with rising PROTAC concentrations, producing an upward curve. In the subsequent 300-s dissociation phase, the signal gradually declined as the molecules dissociated from the protein surface. Generally, higher affinity interactions yield slower dissociation curves (i.e., a plateau), whereas lower affinity interactions show rapid dissociation (Yu et al., 2021).

#### 3.3.2 Comparison of binding affinity

##### 3.3.2.1 Analysis of binding characteristics with CCL5

**FIGURE 6:**
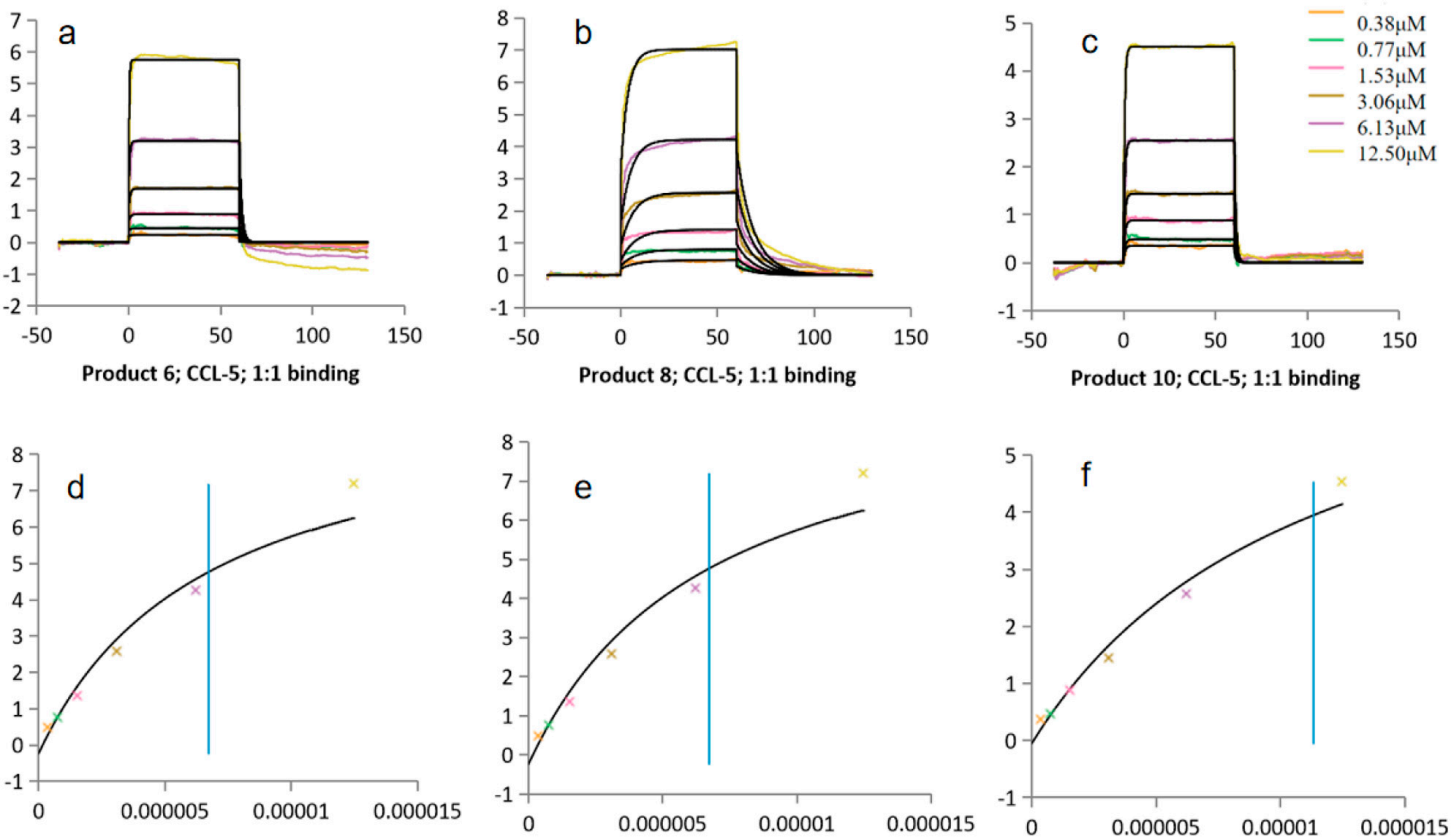
Binding constants (K_D_) of Products 6, 8, 10 with CCL5. **(a–c)** Kinetic fitting curves for products 6, 8 and 10. **(d–f)** Corresponding static binding plots for products 6, 8 and 10.

All three PROTAC molecules exhibited binding affinities for CCL5 in the micromolar to submicromolar range (Table 4; Figure 6). Product 6 showed the lowest K_D_ (4.75 × 10^−6^ M), indicating the highest affinity, followed by Product 8 (9.48 × 10^−6^ M) and then Product 10 (1.13 × 10^−5^ M). These results suggested that while all molecules could bind CCL5, Product 6 was the most effective in capturing the target. This difference might be attributed to the balance between rigidity and flexibility of the linker; an overly short linker (as in Product 10) might restrict conformational freedom, whereas an optimally long linker (as in Product 6) better optimized spatial complementarity and entropic compensation.

**TABLE 4:** Kinetic binding constants (K_D_) of Products 6, 8, 10 with CCL5 and IL-6.

Group	Compound	K_D_ (M)
CCL5	Product 6	4.75 × 10^−6^
CCL5	Product 8	9.48 × 10^−6^
CCL5	Product 10	1.13 × 10^−5^
IL-6	Product 6	8.27 × 10^−6^
IL-6	Product 8	1.54 × 10^−8^
IL-6	Product 10	1.54 × 10^−6^

##### 3.3.2.2 Analysis of binding characteristics with IL-6

**FIGURE 7:**
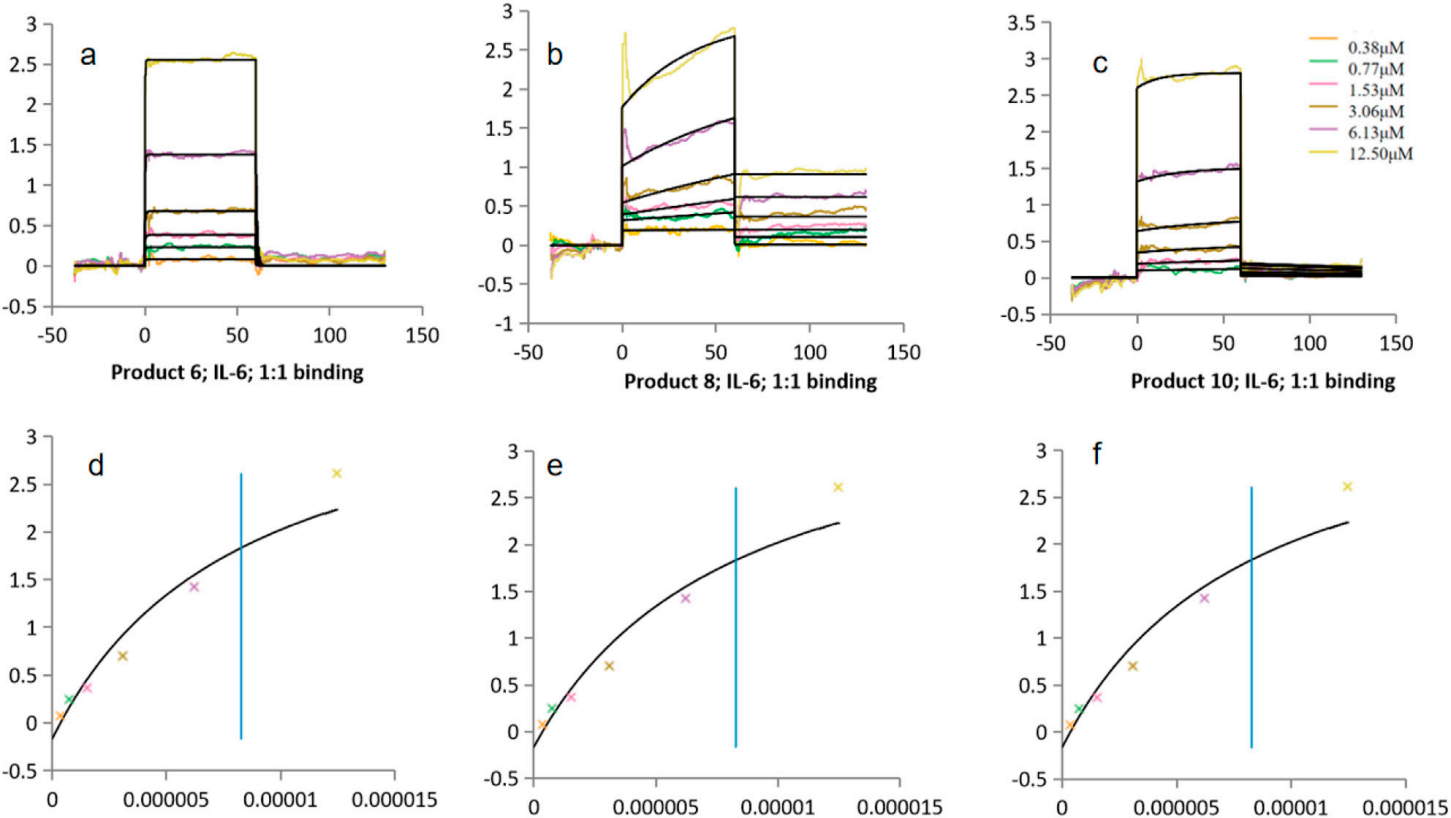
Binding constants (K_D_) of Products 6, 8, 10 with IL-6. **(a–c)** Kinetic fitting curves for products 6, 8 and 10. **(d–f)** Corresponding steady-state binding plots for products 6, 8 and 10. FS control (kinetic fit): K_D_ = 1.35 × 10^−5^ M.

The binding data for IL-6 showed a more complex trend (Figure 7). Product 6 exhibited a K_D_ of 8.27 × 10^−6^ M, whereas Product 10 had a K_D_ of 1.54 × 10^−6^ M—indicating that Product 10 bound IL-6 with somewhat higher affinity. Both values were within a comparable range to those observed with CCL5. In contrast, Product 8 demonstrated an exceptionally low K_D_ of 1.54 × 10^−8^ M, which is 2–3 orders of magnitude lower than the others. However, this value was below the lowest analyte concentration used (0.38 µM = 3.8 × 10^−7^ M), suggesting that nearly all injection concentrations would be saturated in the sensorgram. Such saturation complicated accurate measurement of the dissociation phase; fitting with a simple 1:1 model might therefore underestimate the dissociation rate (k_off_) and yield an overly “optimistic” K_D_. Additional validation—such as varying the flow rate or employing more sophisticated models—is necessary to confirm these results. As this K_D_ lies outside the reliable measurement range, it should be interpreted with caution: sensorgram saturation and mass-transport limitations can reduce the apparent k_off_ and yield an overly optimistic K_D_ from simple 1:1 fits. Although alternative bivalent and heterogeneous-ligand models were tested, they did not improve fit quality, and the 1:1 Langmuir model remained statistically preferred. Future work will include extended dissociation phases, varied flow rates, and more complex fitting models to accurately determine the true kinetic parameters of Product 8.

##### 3.3.2.3 Comparison with the control molecule, FS

FS, used as a negative control, showed no binding to CCL5 and a K_D_ of 13.5 μM with IL-6. This indicates that conventional heparin oligosaccharides do not exhibit strong affinity for these inflammatory mediators. In contrast, the PROTAC molecules, which integrate an E3 ligase binding domain, can form a ternary complex (target protein–PROTAC–E3 ligase) that greatly enhances binding. Notably, the bifunctional architecture of PROTACs not only improves affinity for the target but may also induce a proximity effect that promotes colocalization of the E3 ubiquitin ligase with the target protein, thereby stabilizing the overall interaction—a mechanism entirely absent in FS. Although *in vivo* protein concentrations, conformations, and competing molecules may affect these interactions, the *in vitro* SPR results provide a critical foundation for subsequent cellular and animal studies.

Complementary surface plasmon resonance (SPR) analysis indicated that the anti-inflammatory efficacy of these PROTACs correlates with their high binding affinities for inflammation-related targets such as CCL5 and IL-6. Multiple studies have demonstrated that pomalidomide-based ligands exhibit superior binding and degradation performance compared to lenalidomide analogues. For instance, Yamanaka et al. showed that lenalidomide binds and degrades neosubstrates (e.g., SALL4) less effectively than thalidomide or pomalidomide derivatives (Yamanaka et al., 2023). Consistent with these findings, our pomalidomide-derived PROTACs (Products 6 and 10) displayed higher binding affinities than the lenalidomide analogue (Product 8), likely reflecting improved complementarity at the cereblon (CRBN) interface and enhanced proteasome recruitment. This hypothesis warrants further validation via ubiquitination assays.

### 3.4 Cell-based assays

#### 3.4.1 Overview of cytokine release trends

**FIGURE 8:**
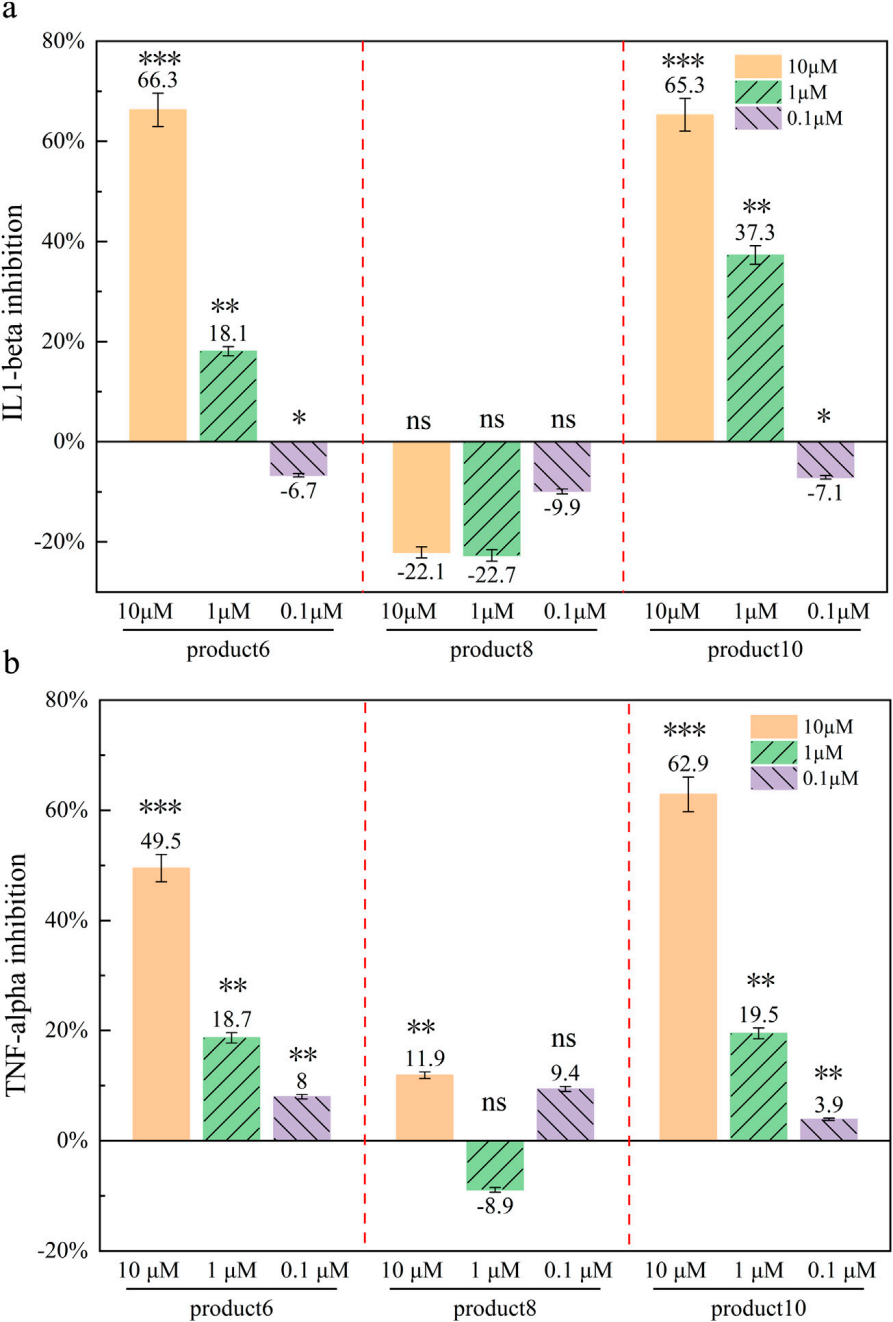
Cytokine inhibition rate of different compounds (%) Inhibition rate of different compounds against different inflammatory factors at different concentrations. **(a)** Inhibition of IL-1β by three PROTAC analogues (6,8,10) at different concentrations; **(b)** Inhibition of TNF-α by three PROTAC analogues (6,8,10) at different concentrations. Data presented as mean ± standard deviations (n = 3). *P* < 0.05 indicated a significant difference compared to the control group. Notes: *p* > 0.05 (ns, not significant): No statistical significance. *p* < 0.05 (*): Significant difference. *p* < 0.01 (**): Highly significant difference. *p* < 0.001 (**): Extremely significant difference.

Under basal conditions, peripheral blood mononuclear cells (PBMCs) maintained low levels of inflammatory cytokines (e.g., IL-1β and TNF-α), reflecting normal secretion under stable growth conditions (Figure 8). Upon lipopolysaccharide (LPS) stimulation, cytokine levels increased markedly (LPS-only group), confirming successful induction of an inflammatory response. In experiments where cells were pretreated with either PROTAC analogs or fondaparinux (FS) followed by LPS addition 2 h later, increasing concentrations of FS and Product 8 did not elicit significant changes in cytokine release. In contrast, both Product 6 and Product 10 demonstrated clear, concentration-dependent suppression of LPS-induced cytokine secretion. Maximum inhibition was observed at 10 μM, with reduced—but still significant—effects at 1 μM, and modest activity at 100 nM.

#### 3.4.2 Comparison among the three PROTAC molecules

At 10 μM, Product 6 significantly inhibited IL-1β and TNF-α levels by 66.3% and 49.5%, respectively. Product 10 also exhibited marked inhibitory effects at 10 μM, with 65.3% inhibition of IL-1β and 62.9% inhibition of TNF-α, although its potency was marginally lower compared to Product 6. By contrast, Product 8 did not display a consistent inhibitory effect—its suppression of IL-1β was not statistically significant (*p* > 0.05, two-tailed t-test) (Supplementary Table S3), and its effects on TNF-α were minimal. Statistical analysis based on mean values and standard deviations confirmed that treatment with 10 µM Product 6 and Product 10 resulted in significant cytokine reduction (*p* < 0.005) (*p* < 0.005, one-way ANOVA with Tukey’s test; *p* < 0.01, unpaired t-test), whereas Product 8 showed limited anti-inflammatory activity under the conditions tested.

At a concentration of 10 μM (≈24 μg/mL, MW = 2,400 Da), Product 10 inhibited LPS-induced IL-1β and TNF-α release by 65.3% and 62.9%, respectively. Similar anti-inflammatory effects have been reported for other PROTACs—for example, the STING-targeting PROTAC SP23 reduced colonic TNF-α and IL-1β levels in a murine colitis model (Xu et al., 2024)—and for natural polyphenols such as resveratrol, which modulate IL-1β and TNF-α secretion in LPS-stimulated cells (Navarro et al., 2015). Our findings are comparable to those observed with 25 μg/mL PE EVOOs/LEVs (Tamburini et al., 2025; Raimondo et al., 2022), which achieved ∼65% IL-1β and ∼70% TNFα inhibition. Furthermore, the inhibitory effect of our PROTACs exhibited a clear concentration dependence, consistent with reports that higher PROTAC concentrations yield greater target degradation (e.g., RIPK2 degraders in THP-1 cells) (Mares et al., 2020). Unlike heterogeneous natural extracts, our PROTACs employ a defined ubiquitin–proteasome–mediated degradation mechanism, ensuring high specificity and reproducibility while permitting further structural optimization to enhance efficacy and minimize off-target effects. Consequently, these synthetic PROTACs offer potential advantages over conventional natural product-based anti-inflammatory agents.

## 4 Conclusion

In conclusion, we successfully demonstrated that integrating Fondaparinux Sodium (FS) into proteolysis-targeting chimera (PROTAC) molecules using a bioprocess engineering approach can overcome challenges in targeting “undruggable” proteins. The direct amidation reaction was systematically optimized—via careful regulation of pH, substrate ratios, and solvent selection—to establish a robust, scalable synthetic platform yielding PROTACs with purity exceeding 99%. Subsequent DEAE-Sephadex ion-exchange chromatography further enhanced product quality by effectively removing impurities. Surface plasmon resonance (SPR) analysis confirmed that the synthesized PROTACs possess nanomolar binding affinities (K_D_ ≈ 10^–6^ M) toward key inflammatory mediators, including RANTES (CCL5) and interleukin-6 (IL-6). Moreover, *in vitro* assays using peripheral blood mononuclear cells (PBMCs) demonstrated that two candidate compounds significantly suppressed LPS-induced interleukin-1β (IL-1β) release in a concentration-dependent manner, achieving inhibition rates of 66.3% and 65.3%, respectively—outperforming FS. The incorporation of FS not only enhances the solubility, *in vivo* distribution, and stability of the PROTACs under physiological conditions but also contributes to their sustained activity. Overall, our work establishes a promising, scalable platform for the production of PROTAC-based anti-inflammatory agents and lays a solid foundation for future *in vivo* validations and mechanistic studies aimed at further optimizing these innovative therapeutic compounds.

## 5 Limitations and future directions

While our FS-based PROTACs have shown strong *in vitro* anti-inflammatory activity, we recognize several barriers to clinical translation that we will address in future work. First, we still need direct mechanistic proof to confirm proteasome-mediated degradation. Second, we were unable to assess efficacy at physiological nanomolar concentrations (10–100 nM) due to resource limitations, so we plan detailed low-concentration dose–response experiments. Third, we have yet to characterize *in vivo* pharmacokinetics and pharmacodynamics in rodents, which will inform absorption, distribution, metabolism, elimination, and dosing. Given the high molecular weight (∼2.4 kDa) and strong anionic character of these molecules, we will explore formulation strategies—such as nanoparticle encapsulation, PEGylation, or prodrug design—to improve bioavailability and half-life. We will also use protein microarrays and acute toxicity studies to profile potential off-target or immunogenic interactions, and ultimately validate long-term safety, biodistribution, and therapeutic efficacy in disease models like rheumatoid arthritis and inflammatory bowel disease.

## Data Availability

The original contributions presented in the study are included in the article/Supplementary Material, further inquiries can be directed to the corresponding authors.

## References

[B1] Abeje Y. E. Wieske L. H. Poongavanam V. Maassen S. Atilaw Y. Cromm P. (2024). Impact of linker composition on VHL PROTAC cell permeability. J. Med. Chem. 68 (1), 638–657. 10.1021/acs.jmedchem.4c02492 39693386 PMC11726670

[B2] Antermite D. Friis S. D. Johansson J. R. Putra O. D. Ackermann L. Johansson M. J. (2023). Late-stage synthesis of heterobifunctional molecules for PROTAC applications via ruthenium-catalysed C‒H amidation. Nat. Commun. 14 (1), 8222. 10.1038/s41467-023-43789-9 38086825 PMC10716378

[B3] Békés M. Langley D. R. Crews C. M. (2022). PROTAC targeted protein degraders: the past is prologue. Nat. Rev. Drug Discov. 21 (3), 181–200. 10.1038/s41573-021-00371-6 35042991 PMC8765495

[B4] Chen X. Soria-Carrera H. Zozulia O. Boekhoven J. (2023). Suppressing catalyst poisoning in the carbodiimide-fueled reaction cycle. Chem. Sci. 14 (44), 12653–12660. 10.1039/D3SC04281B 38020366 PMC10646924

[B5] Chen Y. Liu F. Pal S. Hu Q. (2024). Proteolysis-targeting drug delivery system (ProDDS): integrating targeted protein degradation concepts into formulation design. Chem. Soc. Rev. 53, 9582–9608. 10.1039/D4CS00411F 39171633

[B6] Ciulli A. Trainor N. (2021). A beginner’s guide to PROTACs and targeted protein degradation. Biochem. 43 (5), 74–79. 10.1042/bio_2021_148

[B7] Crowley P. B. (2022). Protein–calixarene complexation: from recognition to assembly. Accounts Chem. Res. 55 (15), 2019–2032. 10.1021/acs.accounts.2c00206 PMC9350911 35666543

[B8] Guedeney N. Cornu M. Schwalen F. Kieffer C. Voisin-Chiret A. S. (2023). PROTAC technology: a new drug design for chemical biology with many challenges in drug discovery. Drug Discov. Today 28 (1), 103395. 10.1016/j.drudis.2022.103395 36228895

[B9] He S. Fang Y. Zhu Y. Ma Z. Dong G. Sheng C. (2024). Drugtamer‐PROTAC conjugation strategy for targeted PROTAC delivery and synergistic antitumor therapy. Adv. Sci. 11 (25), 2401623. 10.1002/advs.202401623 PMC11220662 38639391

[B10] Howard E. L. Goens M. M. Susta L. Patel A. Wootton S. K. (2025). Anti-Drug antibody response to therapeu-tic antibodies and potential mitigation strategies. Biomedicines 13 (2), 299. 10.3390/biomedicines13020299 40002712 PMC11853408

[B11] Jeong Y. Choi J.-M. Kim Y.-K. (2025). Optimizing PROTAC design: a novel approach using exposure scores for linker site selection. In: Proceedings of the 2025 IEEE international conference on big data and smart computing (BigComp), January 15–18, 2025, Kota Kinabalu, Malaysia (IEEE). 9–12. 10.1109/BigComp64353.2025.00011

[B12] Krieger J. Sorrell F. J. Wegener A. A. Leuthner B. Machrouhi‐Porcher F. Hecht M. (2023). Systematic potency and property assessment of VHL ligands and implications on PROTAC design. ChemMedChem. 18 (8), e202200615. 10.1002/cmdc.202200615 36749883

[B13] Kurczewska J. (2022). Recent reports on polysaccharide-based materials for drug delivery. Polymers 14 (19), 4189. 10.3390/polym14194189 36236137 PMC9572459

[B14] Liu Z. Hu M. Yang Y. Du C. Zhou H. Liu C. (2022). An overview of PROTACs: a promising drug discovery paradigm. Mol. Biomed. 3 (1), 46. 10.1186/s43556-022-00112-0 36536188 PMC9763089

[B15] Mares A. Miah A. H. Smith I. E. Rackham M. Thawani A. R. Cryan J. (2020). Extended pharmacodynamic responses observed upon PROTAC-mediated degradation of RIPK2. Commun. Biol. 3 (1), 140. 10.1038/s42003-020-0868-6 32198438 PMC7083851

[B16] Navarro E. Funtikova A. N. Fito M. Schroeder H. (2015). Can metabolically healthy obesity be explained by diet, genetics, and inflammation. Mol. Nutr. Food Res. 59 (1), 75–93. 10.1002/mnfr.201400521 25418549

[B17] Neri-Cruz C. E. Teixeira F. M. E. Gautrot J. E. (2023). A guide to functionalisation and bioconjugation strateg-ies to surface-initiated polymer brushes. Chem. Commun. 59 (49), 7534–7558. 10.1039/D3CC01082A PMC10270241 37194961

[B18] Panja S. Adams D. J. (2022). Chemical crosslinking in “reactive”multicomponent gels. Chem. Commun. 58 (37), 5622–5625. 10.1039/D2CC00919F 35438088

[B19] Raimondo S. Urzì O. Meraviglia S. Di Simone M. Corsale A. M. Rabienezhad Ganji N. (2022). Anti‐inflammatory properties of lemon‐derived extracellular vesicles are achieved through the inhibition of ERK/NF‐κB signalling pathways. J. Cell. Mol. Med. 26 (15), 4195–4209. 10.1111/jcmm.17404 35789531 PMC9344827

[B20] Shute J. K. (2023). Heparin, low molecular weight heparin, and non-anticoagulant derivatives for the treat-ment of inflammatory lung disease. Pharmaceuticals 16 (4), 584. 10.3390/ph16040584 37111341 PMC10141002

[B21] Sincere N. I. Anand K. Ashique S. Yang J. You C. (2023). PROTACs: emerging targeted protein degradati-on approaches for advanced druggable strategies. Molecules 28 (10), 4014. 10.3390/molecules28104014 37241755 PMC10224132

[B22] Sun Y. Jing X. Ma X. Feng Y. Hu H. (2020). Versatile types of polysaccharide-based drug delivery systems: from strategic design to cancer therapy. Int. J. Mol. Sci. 21 (23), 9159. 10.3390/ijms21239159 33271967 PMC7729619

[B23] Syahputra E. W. Lee H. Cho H. Park H. J. Park K.-S. Hwang D. (2025). PROTAC delivery strategies for overcoming physicochemical properties and physiological barriers in targeted protein degradation. Pharmaceutics 17 (4), 501. 10.3390/pharmaceutics17040501 40284496 PMC12030311

[B24] Tamburini B. Di Liberto D. Pratelli G. Rizzo C. Barbera L. L. Lauricella M. (2025). Extra virgin olive oil polyphenol-enriched extracts exert antioxidant and anti-inflammatory effects on peripheral blood mononuclear cells from rheumatoid arthritis patients. Antioxidants 14 (2), 171. 10.3390/antiox14020171 40002358 PMC11851824

[B25] Tan M. Li X. Cheng L. Long X. Cao G. Yu S. (2025). Augmenting protein degradation capacity of PROTAC through energy metabolism regulation and targeted drug delivery. Adv. Mater. 37 (1), 2412837. 10.1002/adma.202412837 39491551

[B26] Todaro B. Ottalagana E. Luin S. Santi M. (2023). Targeting peptides: the new generation of targeted drug delivery systems. Pharmaceutics 15 (6), 1648. 10.3390/pharmaceutics15061648 37376097 PMC10300729

[B27] Vetma V. O’Connor S. Ciulli A. (2024). Development of PROTAC degrader drugs for cancer. Annu. Rev. Cancer Biol. 9, 119–140. 10.1146/annurev-cancerbio-061824-105806

[B28] Wang S. He F. Tian C. Sun A. (2024). From PROTAC to TPD: advances and opportunities in targeted protein degradation. Pharmaceuticals 17 (1), 100. 10.3390/ph17010100 38256933 PMC10818447

[B29] Xu S. Peng Y. Yang K. Liu S. He Z. Huang J. (2024). PROTAC based STING degrader attenuates acute colitis by inhibiting macrophage M1 polarization and intestinal epithelial cells pyroptosis mediated by STING-NLRP3 axis. Int. Immunopharmacol. 141, 112990. 10.1016/j.intimp.2024.112990 39223062

[B30] Yamanaka S. Furihata H. Yanagihara Y. Taya A. Nagasaka T. Usui M. (2023). Lenalidomide derivatives and proteolysis-targeting chimeras for controlling neosubstrate degradation. Nat. Commun. 14 (1), 4683. 10.1038/s41467-023-40385-9 37596276 PMC10439208

[B31] Yu M. Zhang T. Li J. P. Tan Y. (2021). Elucidating the binding mode between heparin and inflammatory c-ytokines by molecular modeling. ChemistryOpen 10 (10), 966–975. 10.1002/open.202100135 34596979 PMC8485826

[B32] Zhong G. Chang X. Xie W. Zhou X. (2024). Targeted protein degradation: advances in drug discovery and clinical practice. Signal Transduct. Target. Ther. 9 (1), 308. 10.1038/s41392-024-02004-x 39500878 PMC11539257

