# Supplementary material for: Design, synthesis, and anti-inflammatory potential of PROTAC drug molecules based on fondaparinux sodium
Source: Front Bioeng Biotechnol. 2025 Jul 7;13:1597344. doi: 10.3389/fbioe.2025.1597344 (PMC12277314; doi:10.3389/fbioe.2025.1597344)
Supplement: Supplementary file 1 [file DataSheet1.docx]

Supplementary Material

# Supplementary Data

**Figure S1** LC-MS analysis for compound 1


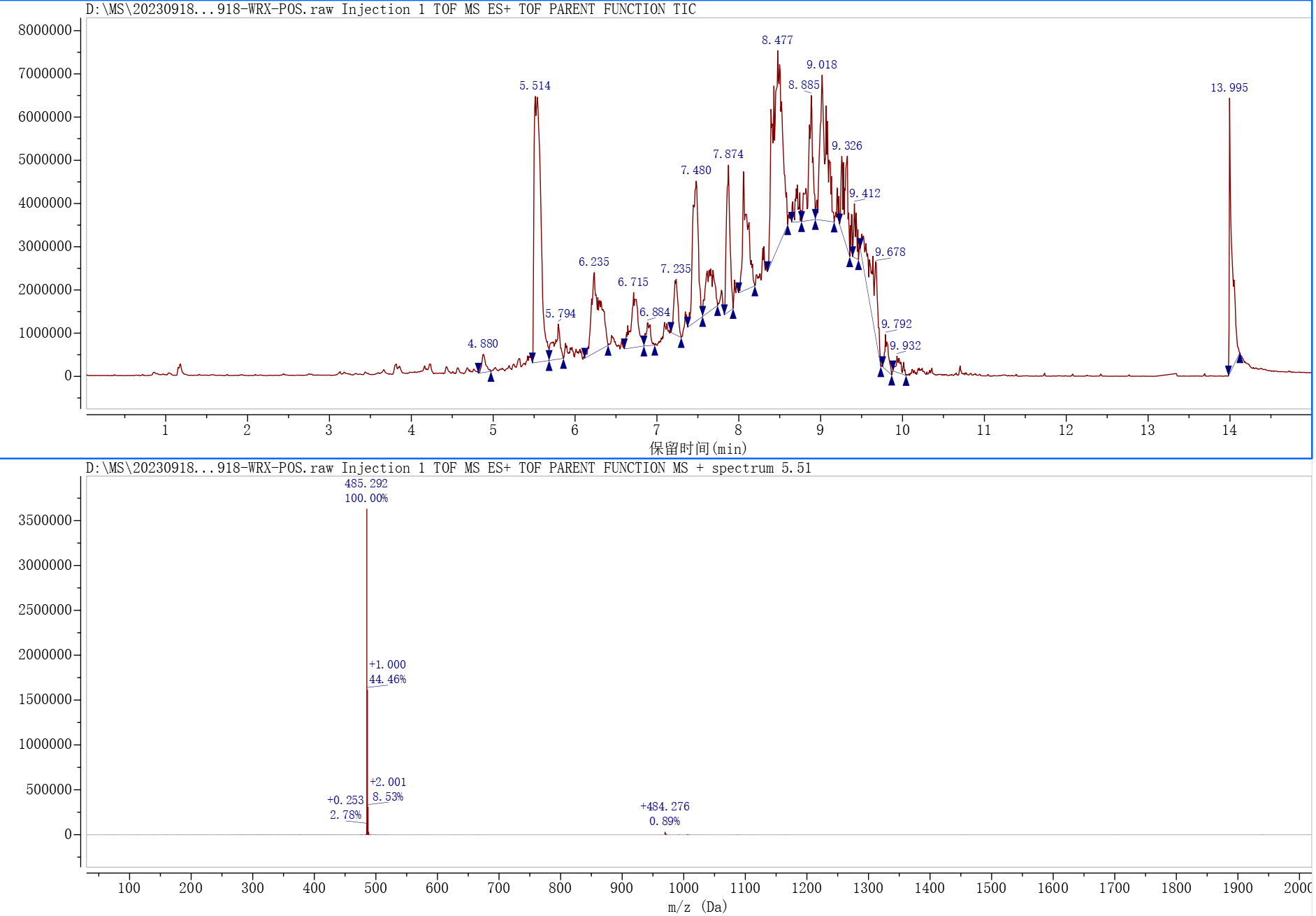

**Figure S2** LC-MS analysis for compound 2


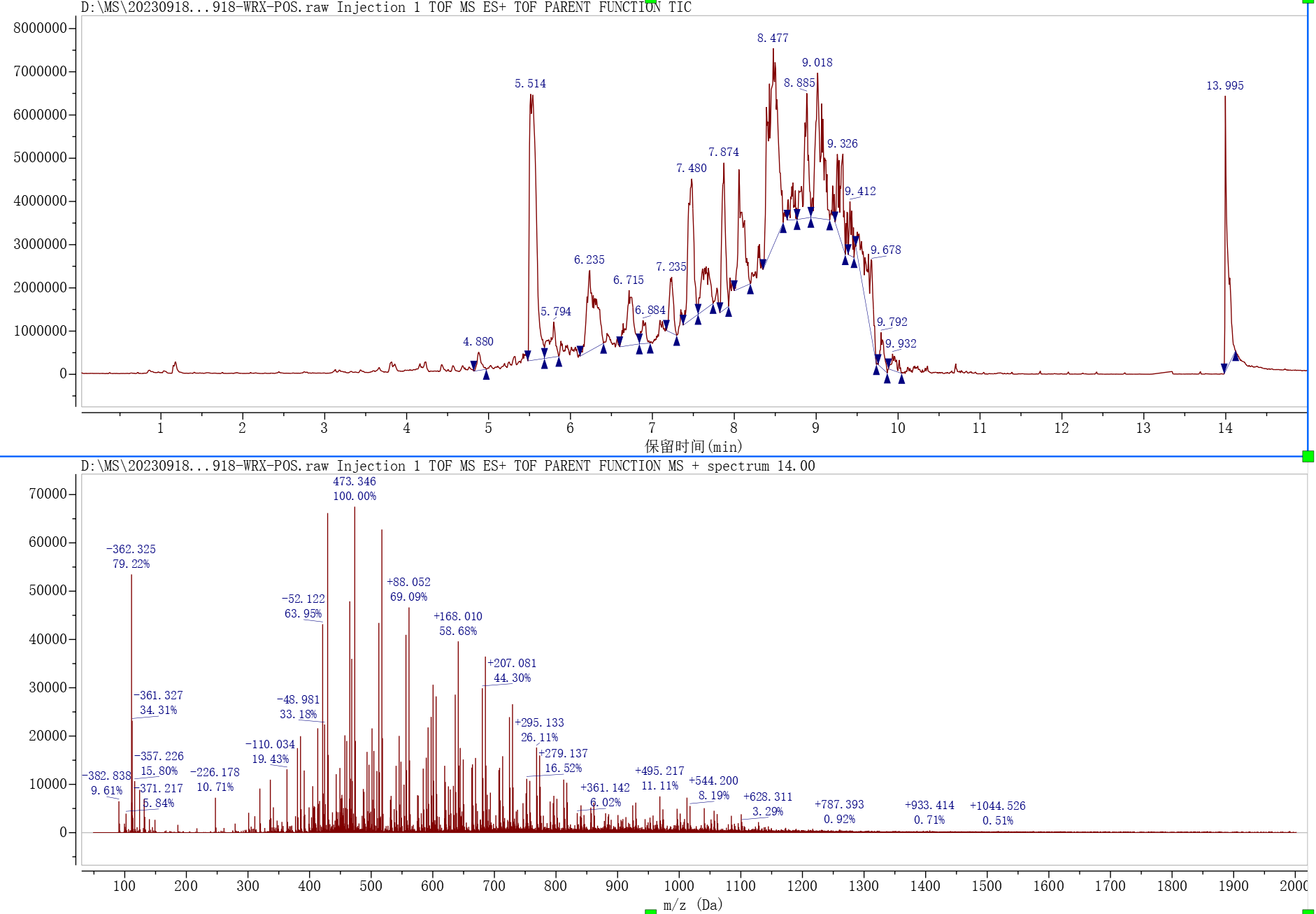


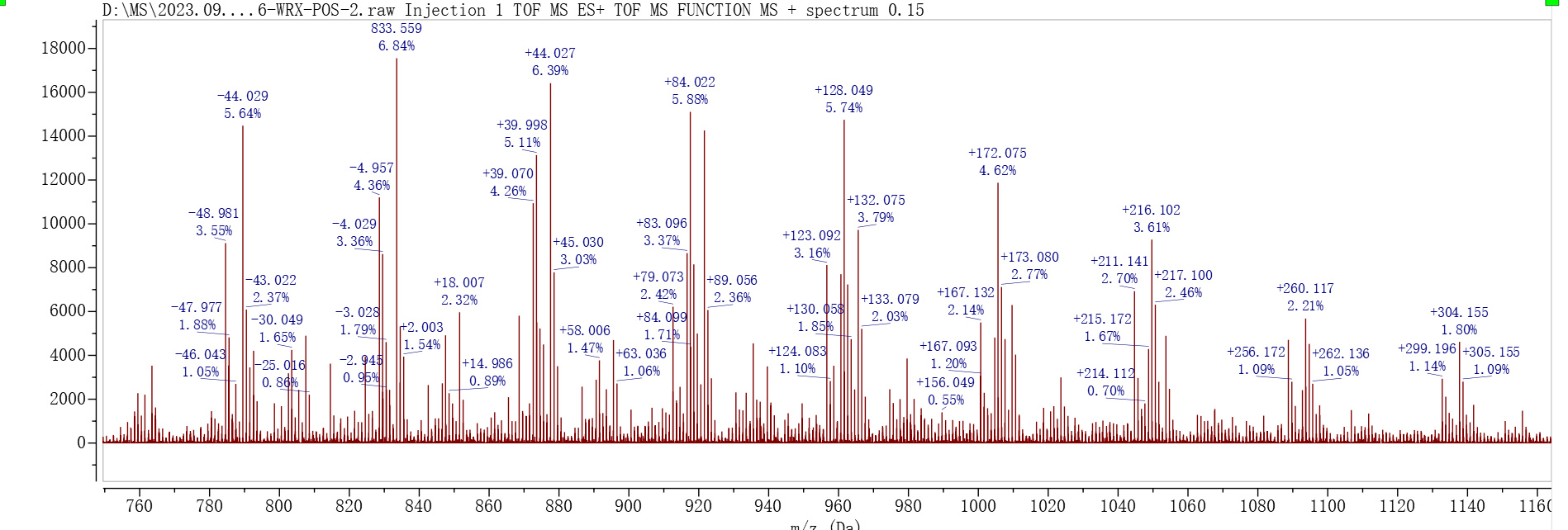

**Table S1** LC-MS analysis of substances under different reaction conditions

| compound | m/z | RT（min) | peak area | | | | | | | | | | | | | | | |
| --- | --- | --- | --- | --- | --- | --- | --- | --- | --- | --- | --- | --- | --- | --- | --- | --- | --- | --- |
|  |  |  | A1 | A2 | A3 | A4 | A5 | A6 | A7 | A8 | A9 | A10 | A11 | A12 | A13 | A14 | A15 | A16 |
| product 6 | 746.7629 z=3  447.6569 z=4  559.8230 z=2 | 28.88 | 0 | 39588 | 0 | 1547930 | 2152596 | 2359809 | 5974258 | 159297 | 15058 | 33652 | 1748 | 2541335 | 1230871 | 3908607 | 1499076 | 50095 |
| intermediates 3 | 675.7522 z=3 | 29.80-30.22 | 0 | 0 | 0 | 0 | 254999 | 465336 | 652805 | 50342 | 0 | 109262 | 78581 | 156045 | 180763 | 351566 | 113457 | 11119 |
| byproduct 5(a,b) | 374.0199 z=5  467.7768 z=4  624.0372 z=3  936.5608 z=2 | 30.9 | 22139 | 75048 | 2152500 | 3434117 | 4371917 | 10346158 | 6661654 | 197765 | 0 | 13979 | 0 | 1510523 | 1352938 | 1045965 | 269136 | 18670 |
| intermediates 11 | 604.7380 z=3 | 31.68 | 320830 | 0 | 0 | 36529 | 32539 | 145474 | 0 | 0 | 0 | 2688 | 1379 | 0 | 0 | 0 | 0 | 0 |
| intermediates 4 | 331.4144 z=5  414.5161 z=4  553.0239 z=3  830.0395 z=2 | 32.67 | 647665 | 356478 | 4924361 | 2813478 | 2714471 | 8045246 | 1003141 | 22779 | 537349 | 163873 | 182487 | 188902 | 148019 | 157917 | 91091 | 71139 |
| other | 818.1451 z=3  647.7234 z=3  671.4031 z=3 ... | _ | 883691 | 1388390 | 7068586 | 4067051 | 3462405 | 12475502 | 7505166 | 144258 | 0 | 10654 | 0 | 1841106 | 1474773 | 831040 | 215281 | 24485 |
| product8* | 708.7487 z=3 | 28.65 | - | - | - | - | - | - | - | 85477 | - | - | - | - | - | - | 82229 | - |
| product7*(a,b) | 605.0278 z=3 | 31.52 | - | - | - | - | - | - | - | 1291431 | - | - | - | - | - | - | 261631 | - |
| product **10 | 728.0760 z=3 | 31 | - | - | - | - | - | - | - | 120219 | - | - | - | - | - | - | 147732 | - |
| product9**(a,b) | 614.6917 z=3 | 28.65 | - | - | - | - | - | - | - | 178151 | - | - | - | - | - | - | 377519 | - |
| intermediate4* | 553.0303 z=3 | 32.59 | - | - | - | - | - | - | - | 23287 | - | - | - | - | - | - | 20006 | - |
| intermediate4** | 553.0303 z=3 | 32.59 | - | - | - | - | - |  | - | 10088 | - | - | - | - | - | - | 9877 | - |

a,b refer to regioisomers where the R1 and R2 groups are swapped, and the yield represents the combined yield of both (as they are difficult to separate).

*Only the Pomalidomide molecule was replaced with Lenalidomide under otherwise identical conditions.

**Only the Pomalidomide molecule was replaced with Thalidomide under otherwise identical condition

**Table S2** LC-MS identification of the products isolated at different sodium chloride concentrations with optimized conditions

| compound | m/z | RT（min) | gradient of NaCl concentration(M) | | | | | |
| --- | --- | --- | --- | --- | --- | --- | --- | --- |
|  |  |  | 0.7 | 0.75 | 0.8 | 0.85 | 0.9 | 0.925 |
| product 6 | 746.7629 z=3  447.6569 z=4  559.8230 z=2 | 28.88 | 8470754 | 14945 | 54552 | 1679998 | 292095 | 1803135 |
| intermediates 3 | 675.7522 z=3 | 29.80-30.22 | 49730 | 0 | 198546 | 0 | 0 | 14479 |
| byproduct 5(a,b) | 374.0199 z=5  467.7768 z=4  624.0372 z=3  936.5608 z=2 | 30.9 | 5450483 | 186488 | 662590 | 250496 | 0 | 236069 |
| intermediates 11 | 604.7380 z=3 | 31.68 | 0 | 0 | 0 | 0 | 0 | 0 |
| intermediates 4 | 331.4144 z=5  414.5161 z=4  553.0239z=3  830.0395 z=2 | 32.67 | 69322 | 3554 | 108208 | 0 | 0 | 6690 |
| other | 818.1451 z=3  647.7234 z=3  671.4031 z=3 ... | _ | 5817710 | 225455 | 1037062 | 329207 | 2449 | 64625 |
| fondaparinux sodium | 752.4684 z=2  501.3149 z=3  375.7305 z=4 | 33.85 | 3063806 | 106514 | 150132 | 1297 | 0 | 41016 |

**Figure S3** LC-MS characterization of compounds 3-10


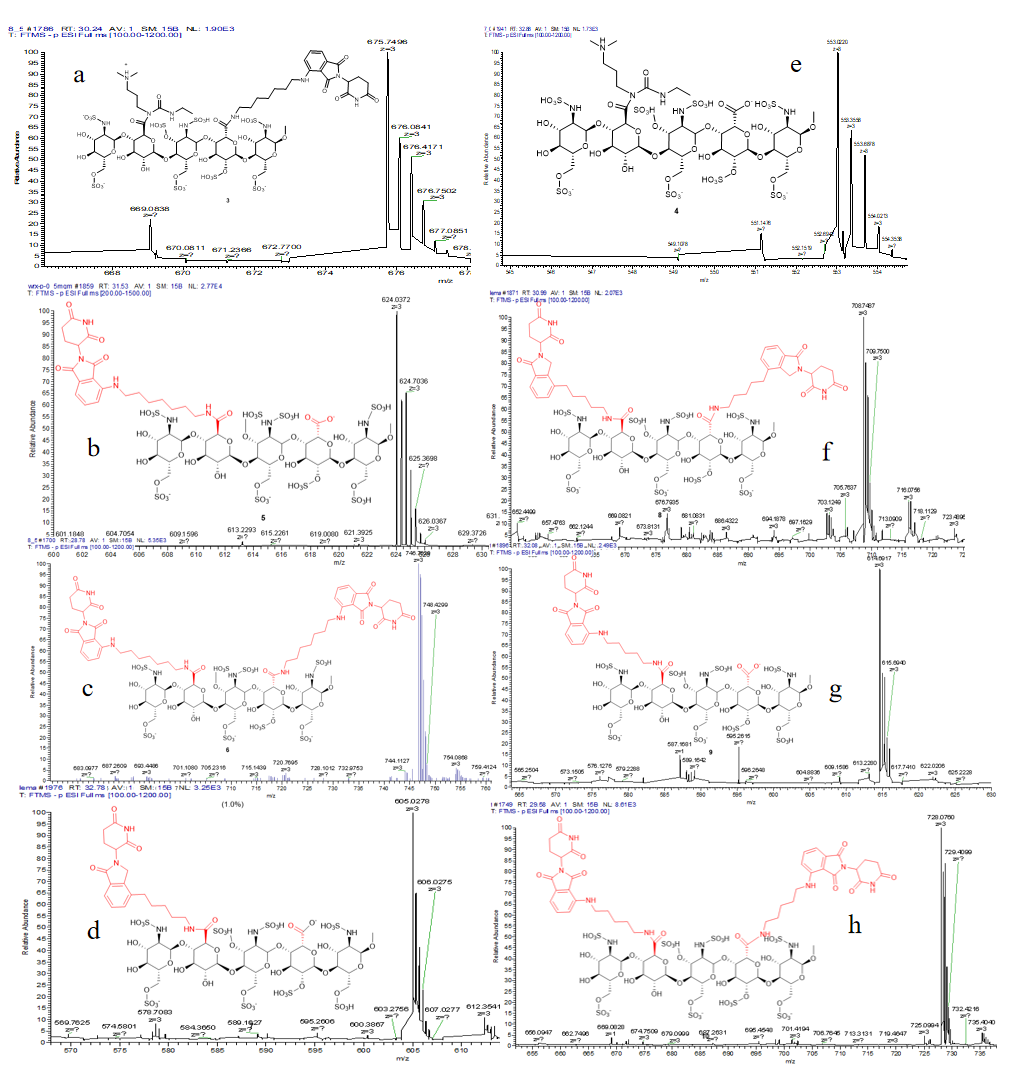


(a) Intermediate 3 mass spectrum (ESI anion mode, m/z 100–1200) [M-3H]^3-^ m/z = 675.7522, (b) 5(a,b) mass spectrum (ESI anion mode, m/z 100–1200) [M−3H]^3−^ m/z =624.0372,(c) 6 mass spectrum (ESI anion mode, m/z 100–1200) [M−3H]^3−^ m/z =447.6569,(d) 7(a,b) mass spectrum (ESI anion mode, m/z 100–1200) [M−3H]^3−^ m/z = 605.0278,(e) Intermediate 4 mass spectrum (ESI anion mode, m/z 100–1200) [M-3H]^3-^ m/z = 553.0239, (f) 8 mass spectrum (ESI anion mode, m/z 100–1200) [M−3H]^3−^ m/z = 708.7487,(g) 9(a,b) mass spectrum (ESI anion mode, m/z 100–1200) [M−3H]^3−^ m/z = 614.6917,(h) 10 mass spectrum (ESI anion mode, m/z 100–1200) [M−3H]^3−^ m/z = 728.0760

**
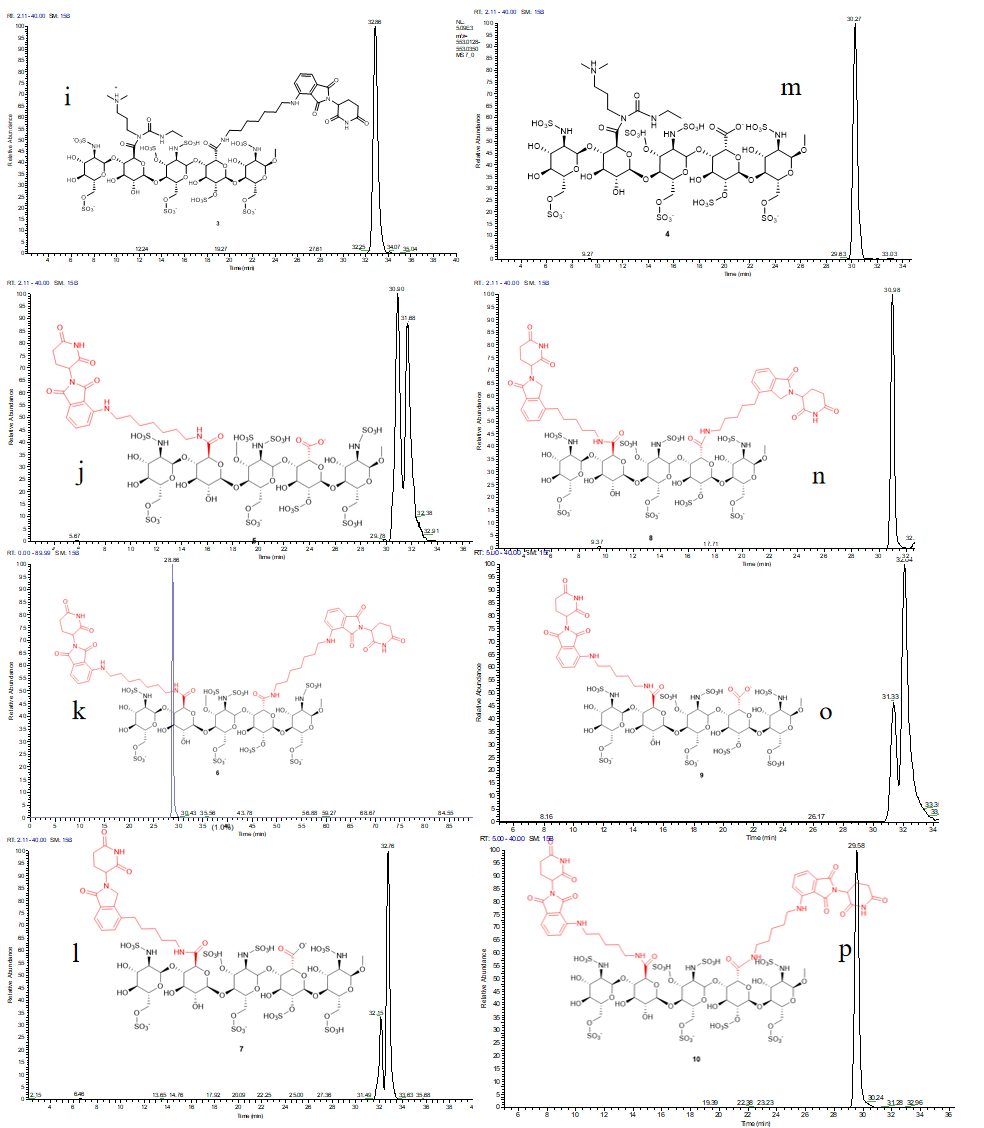
**

Retention times (RT) for monitored compounds are as follows(i)intermediate 3 (RT 29.80–30.22 min),(j)byproduct 5 (a,b) (RT 30.90 min), (k)product 6 (RT 28.88 min), (l)product 7 (a,b) (RT 31.52 min), (m)intermediate 4 (RT 32.67 min),(n)product 8 (RT 28.65 min), (o)product 9 (a,b) (RT 28.65 min), (p)product 10 (RT 31.00 min).

**Figure S4** The ^1^H NMR identification of (a) product 6 and (b) ligand standards


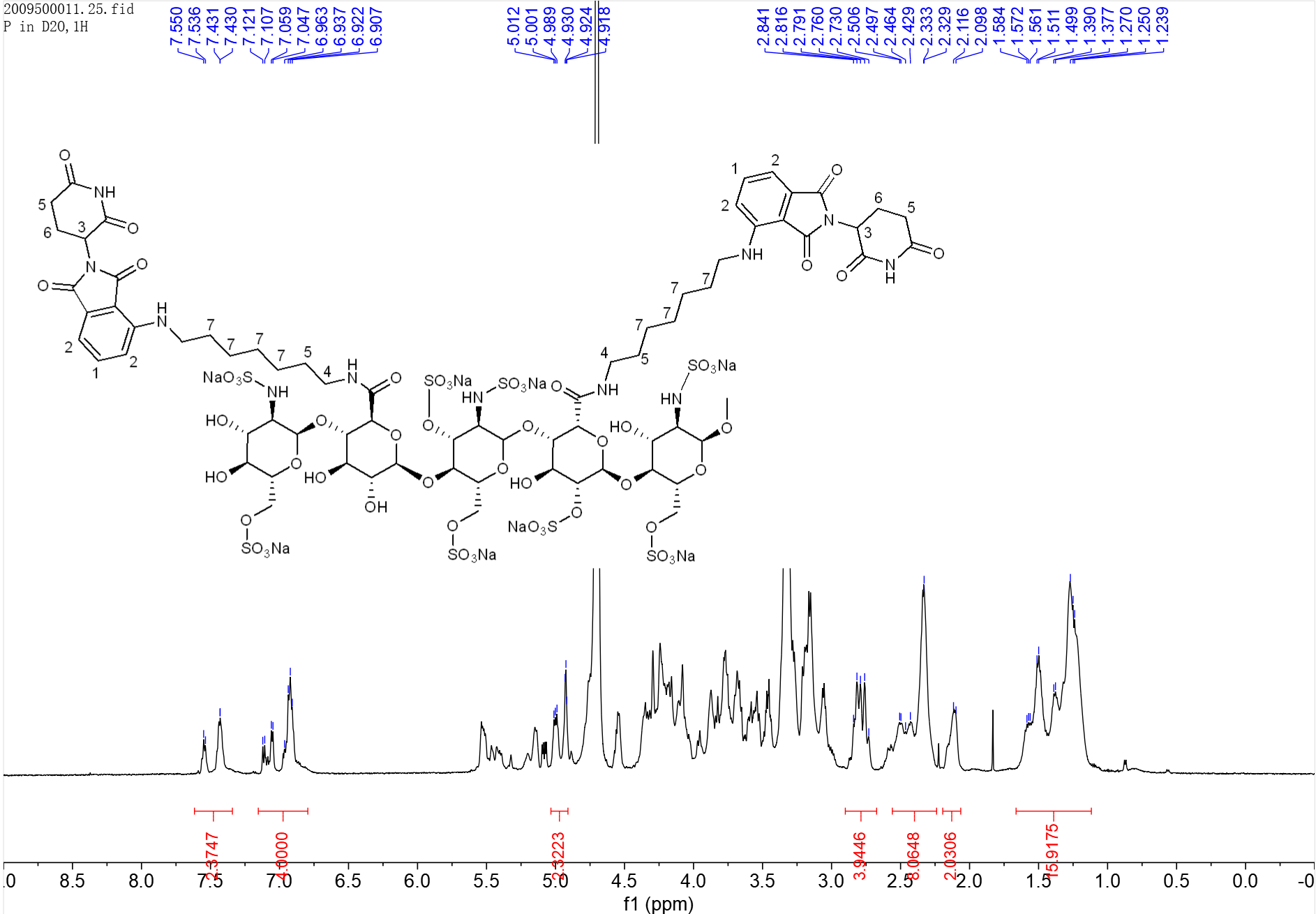


a


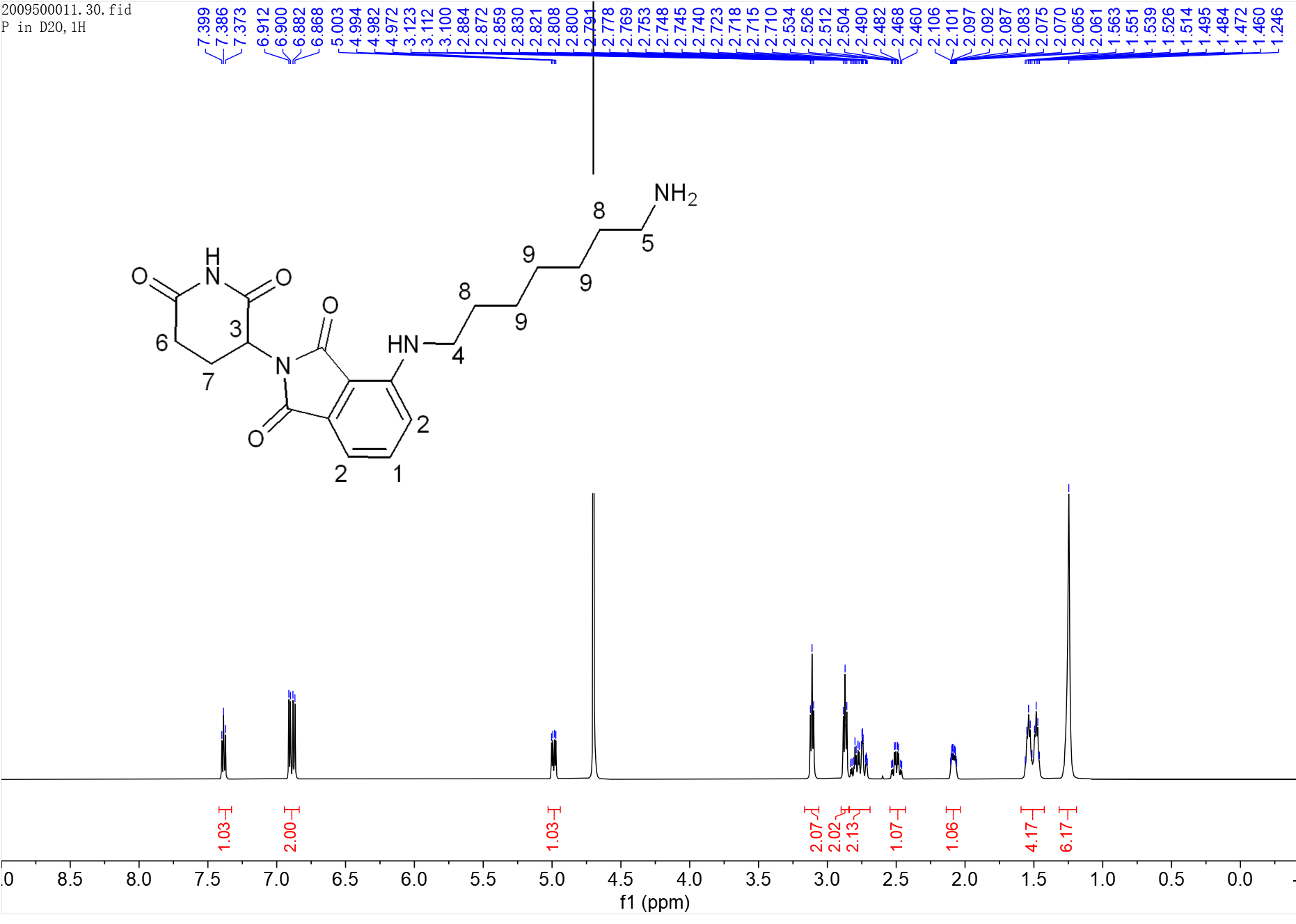


b

(a)^1^H NMR (600 MHz, Deuterium Oxide) δ 7.38 - 7.61 (m, 2H, H1), 6.79 - 7.16 (m, 4H, H2), 4.90 - 5.03 (m, 2H, H3), 2.68 - 2.92 (m, 4H, H4), 2.24 - 2.55 (m, 8H, H5), 2.06 - 2.19 (m, 2H, H6), 1.09 - 1.65 (m, 16H, H7)

(b)^1^H NMR (600 MHz, Deuterium Oxide) δ7.38 (t, J = 8.2 Hz, 1H, H1), 6.89 (dd, J = 18.6, 7.9 Hz, 2H, H2), 4.99 (dd, J = 12.9, 5.6 Hz, 1H, H3), 3.11 (t, J = 7.1 Hz, 2H, H4), 2.87 (t, J = 7.6 Hz, 2H, H5), 2.85 - 2.68 (m, 2H, H6), 2.50 (qd, J = 13.1, 4.9 Hz, 1H, H7-1), 2.11 - 2.06 (m, 1H, H7-2), 1.51 (dq, J = 32.5, 7.2 Hz, 4H, H8), 1.25 (s, 6H, H9)

**Figure S5** The ^1^H NMR identification of (a) product 8 and (b) ligand standards


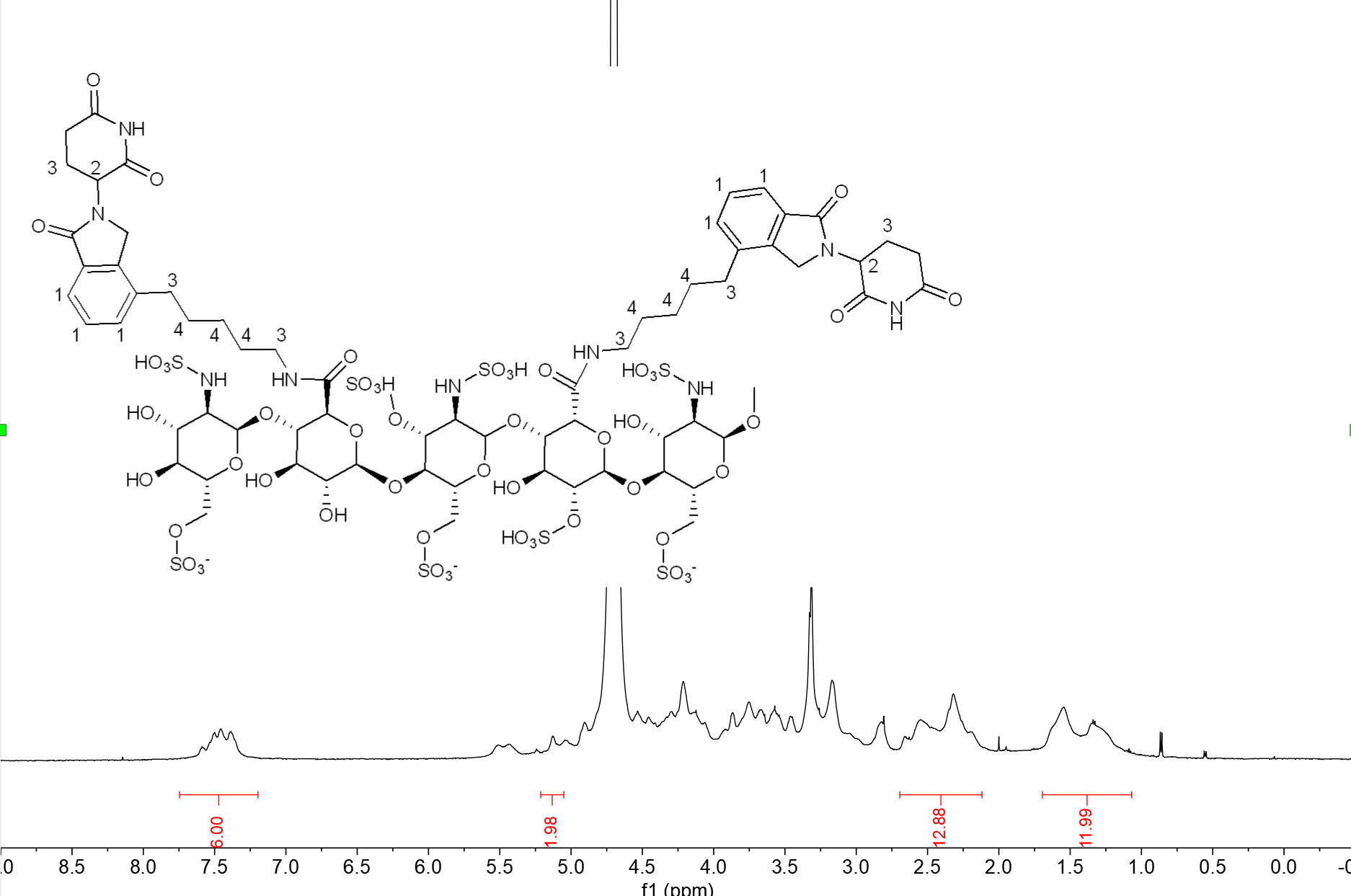


a

^
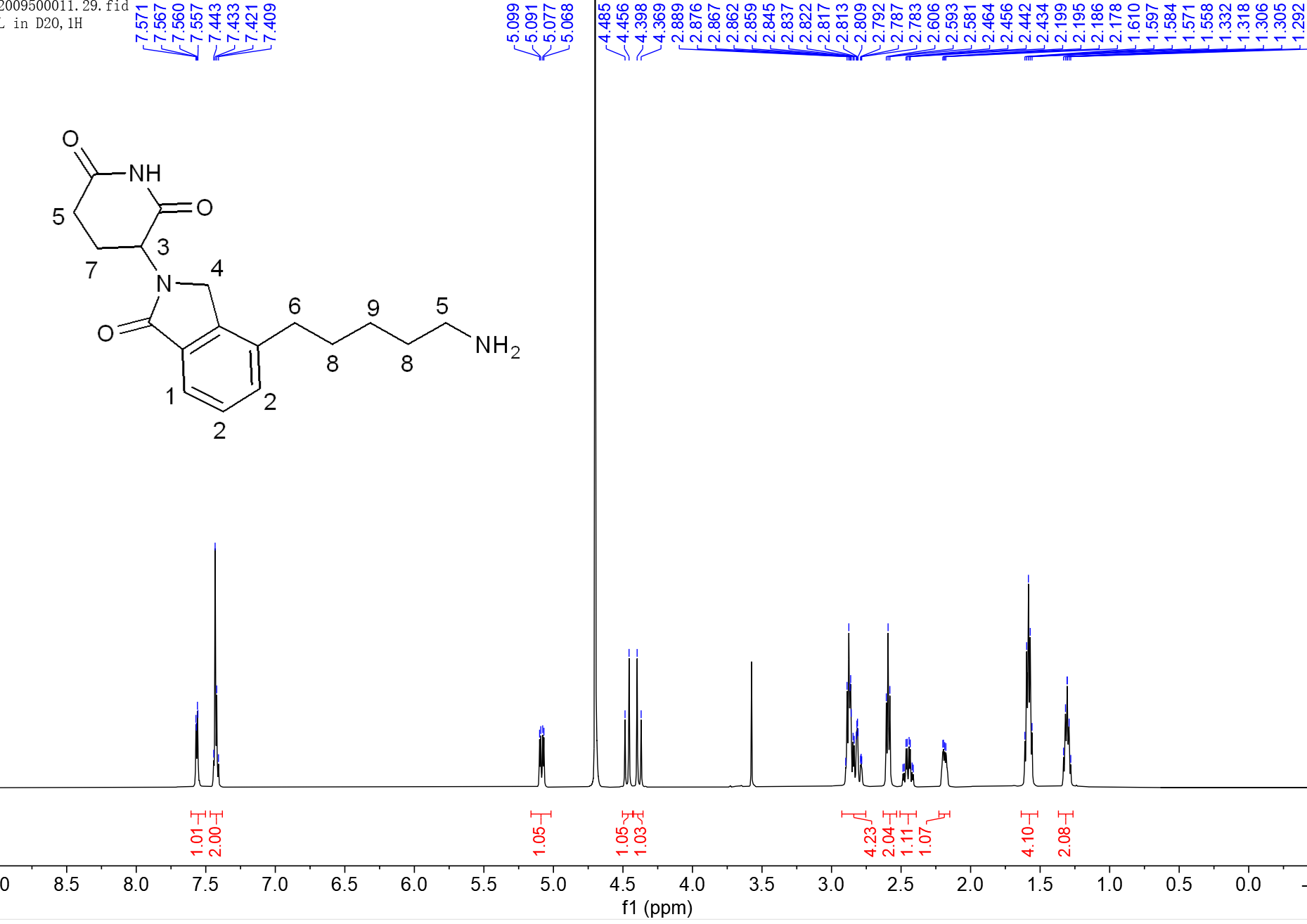
^

b

(a)^1^H NMR (600 MHz, Deuterium Oxide) δ 7.19 - 7.68 (m, 6H, H1), 4.98 - 5.18 (m, 2H, H2), 2.12 -2.68 (m, 12H, H3), 1.07 - 1.70 (m, 12H, H4)

(b)^1^H NMR (600 MHz, Deuterium Oxide) δ7.56 (dd, J = 6.4, 2.0 Hz, 1H, H1), 7.39 - 7.45 (m, 2H, H2), 5.08 (dd, J = 13.4, 5.2 Hz, 1H, H3), 4.47 (d, J = 17.3 Hz, 1H, H4-1), 4.38 (d, J = 17.3 Hz, 1H, H4-2), 2.91 - 2.77 (m, 4H, H5), 2.59 (t, J = 7.7 Hz, 2H, H6), 2.45 (qd, J = 13.1, 5.2 Hz, 1H, H7-1), 2.19 (dd, J = 9.2, 3.9 Hz, 1H, H7-2), 1.58 (p, J = 7.7 Hz, 4H, H8), 1.31 (p, J = 7.7 Hz, 2H, H9)

**Figure S6** The ^1^H NMR identification of (a) product 10 and (b) ligand standards

a


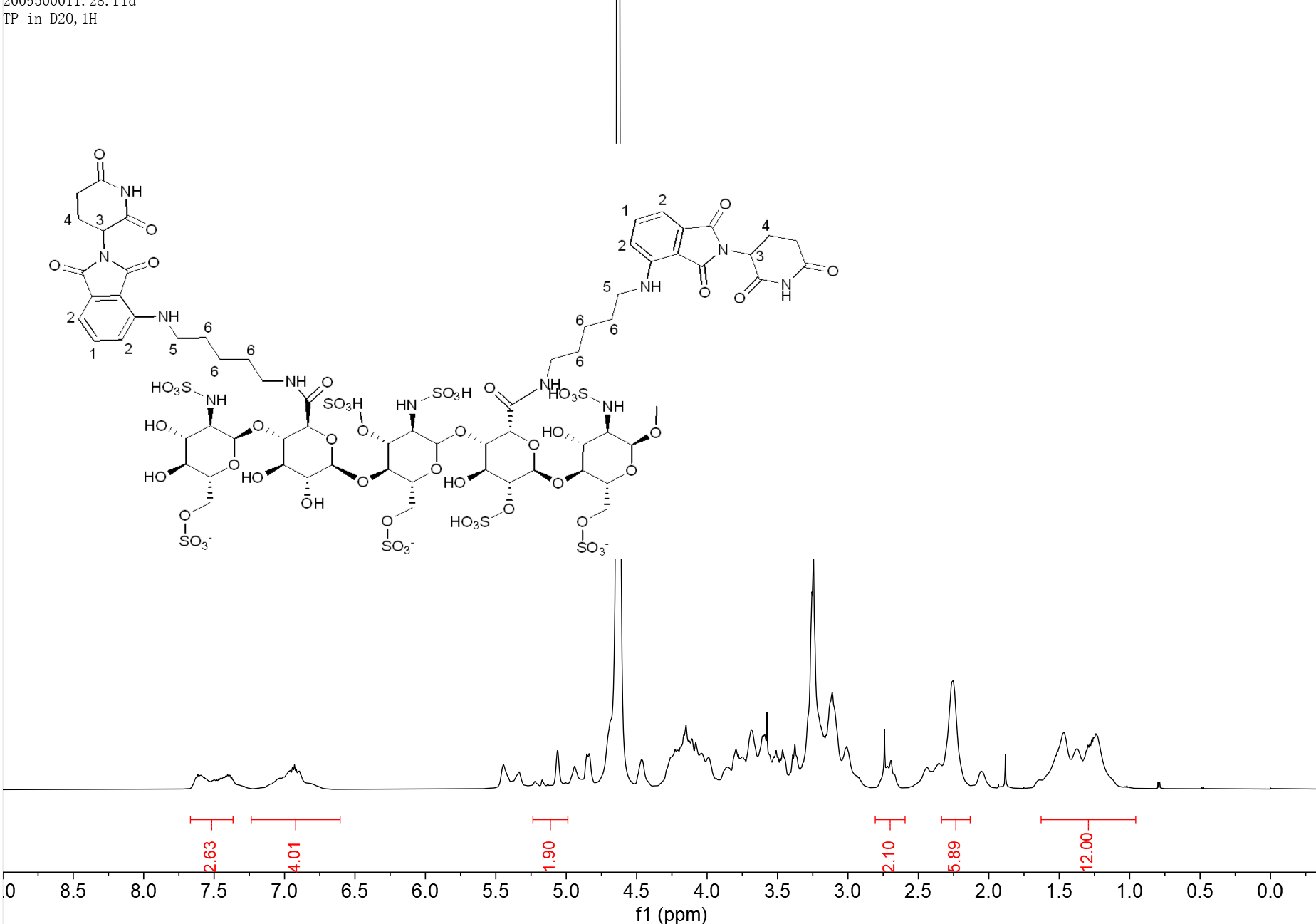


^
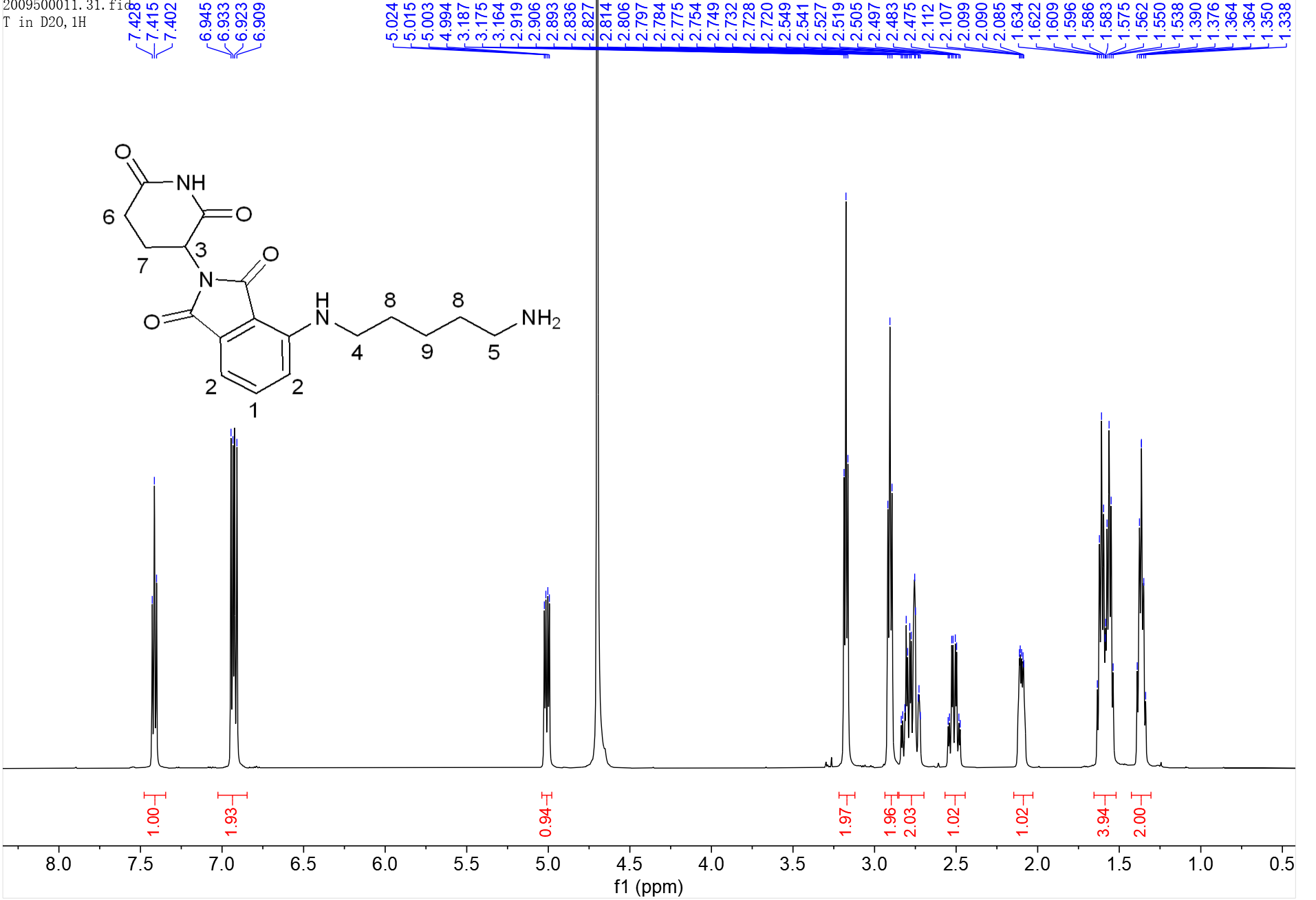
^

b

（a）^1^H NMR (600 MHz, Deuterium Oxide) δ7.36 - 7.66 (m, 2H, H1), 6.60 - 7.23 (m, 4H, H2), 4.99 - 5.23 (m, 2H, H3), 2.58 - 2.83 (m, 2H, H4-1), 2.13 -2.33 (m, 6H, H4-2& H5), 1.00 - 1.63(m, 12H, H6)

(b)^1^H NMR (600 MHz, Deuterium Oxide) δ 7.41 (t, J = 8.0 Hz, 1H, H1), 6.93 (dd, J = 13.9, 7.8 Hz, 2H, H2), 5.01 (dd, J = 12.9, 5.6 Hz, 1H, H3), 3.18 (t, J = 7.0 Hz, 2H, H4), 2.91 (t, J = 7.7 Hz, 2H, H5), 2.77 (ddd, J = 28.7, 13.1, 4.0 Hz, 2H, H6), 2.51 (qd, J = 13.1, 5.0 Hz, 1H, H7-1), 2.07 - 2.13 (m, 1H, H7-2), 1.59 (dp, J = 27.3, 7.5 Hz, 4H, H8), 1.36 (dq, J = 15.4, 7.7, 7.0 Hz, 2H, H9)

**Table S3** Significance Analysis of product 6,7,8 at Different Concentrations on Inflammatory Factors

| Compound | Concentration (μM) | IL-1β Inhibition Significance | TNF-α Inhibition Significance |
| --- | --- | --- | --- |
| product8 | 100 nM | ns (*p* = 0.45) | ns (*p* = 0.19) |
|  | 1 μM | ns (*p* = 0.52) | ns (*p* = 0.07) |
|  | 10 μM | ns (*p* = 0.08) | * (*p* = 0.04) |
| product10 | 100 nM | * (*p* = 0.02) | ** (*p* = 0.006) |
|  | 1 μM | ** (*p* = 0.004) | ** (*p* = 0.002) |
|  | 10 μM | *** (*p* < 0.001) | *** (*p* < 0.001) |
| product6 | 100 nM | * (*p* < 0.05) | ** (*p* = 0.006) |
|  | 1 μM | ** (*p* < 0.01) | ** (*p*= 0.002) |
|  | 10 μM | *** (*p* < 0.001) | *** (*p* < 0.001) |

Notes:

*p* > 0.05 (ns, not significant): No statistical significance.

*p* < 0.05 (*): Significant difference.

*p* < 0.01 (**): Highly significant difference.

*p* < 0.001 (**): Extremely significant difference.

Summary

Product 8 exhibits weak effects, showing only mild inhibition of TNF-α at 10 μM, with no significant effect on IL-1β.

Product 10 shows a significant inhibitory effect on inflammatory factors starting from 100 nM, displaying a concentration-dependent trend, with the strongest effect at 10 μM.

Product 6 demonstrates significant inhibition in all inflammatory factor assays, showing noticeable effects from 100 nM, with an overall stronger inhibition than Product 10.
